# Interleukin 17 signaling supports clinical benefit of dual CTLA-4 and PD-1 checkpoint inhibition in melanoma

**DOI:** 10.1038/s43018-023-00610-2

**Published:** 2023-07-31

**Authors:** Renáta Váraljai, Lisa Zimmer, Yahya Al-Matary, Paulien Kaptein, Lea J. Albrecht, Batool Shannan, Jan C. Brase, Daniel Gusenleitner, Teresa Amaral, Nina Wyss, Jochen Utikal, Lukas Flatz, Florian Rambow, Hans Christian Reinhardt, Jenny Dick, Daniel R. Engel, Susanne Horn, Selma Ugurel, Wiebke Sondermann, Elisabeth Livingstone, Antje Sucker, Annette Paschen, Fang Zhao, Jan M. Placke, Jasmin M. Klose, Wolfgang P. Fendler, Daniela S. Thommen, Iris Helfrich, Dirk Schadendorf, Alexander Roesch

**Affiliations:** 1 Department of Dermatology, University Hospital Essen, West German Cancer Center, University Duisburg-Essen and the German Cancer Consortium (DKTK), Essen, Germany; 2 Division of Molecular Oncology and Immunology, the Netherlands Cancer Institute, Amsterdam, the Netherlands; 3 Novartis Pharma AG, Basel, Switzerland; 4 Novartis Institutes for BioMedical Research, Inc., Cambridge, MA USA; 5 Department of Dermatology, University Hospital of Tübingen, Tübingen, Germany; 6 Institute of Immunobiology, Kantonsspital St. Gallen, Switzerland, Switzerland; 7 Skin Cancer Unit, German Cancer Research Center (DKFZ), Heidelberg, Germany; 8 Department of Dermatology, Venereology and Allergology, University Medical Center Mannheim, Ruprecht Karls University of Heidelberg, Mannheim, Germany; 9 DKFZ Hector Cancer Institute at the University Medical Center Mannheim, Mannheim, Germany; 10 Department of Applied Computational Cancer Research, Institute for AI in Medicine (IKIM), University Hospital Essen, Essen, Germany; 11 Department of Hematology and Stem Cell Transplantation, University Hospital Essen, Essen, Germany; 12 Center for Medical Biotechnology (ZMB), University of Duisburg-Essen, Essen, Germany; 13 Department of Immunodynamics, Institute of Experimental Immunology and Imaging, University Hospital Essen, Essen, Germany; 14 Rudolf Schönheimer Institute of Biochemistry, Medical Faculty, University of Leipzig, Leipzig, Germany; 15 Department of Nuclear Medicine, University Hospital Essen, University of Duisburg-Essen, Essen, Germany; 16 Department of Dermatology and Allergology, Ludwig Maximilian University Munich, Munich, Germany; 17 NCT West, Campus Essen and University Alliance Ruhr, Research Center One Health, University Duisburg-Essen, Essen, Germany

**Keywords:** Melanoma, Cancer immunotherapy, Tumour immunology, Cancer

## Abstract

Recent studies suggest that *BRAF*^V600^-mutated melanomas in particular respond to dual anti-programmed cell death protein 1 (PD-1) and anti-cytotoxic T lymphocyte-associated protein 4 (CTLA-4) immune checkpoint inhibition (ICI). Here we identified an over-representation of interleukin (IL)-17–type 17 helper T (T_H_17) gene expression signatures (GES) in *BRAF*^V600^-mutated tumors. Moreover, high baseline IL-17 GES consistently predicted clinical responses in dual-ICI-treated patient cohorts but not in mono anti-CTLA-4 or anti-PD-1 ICI cohorts. High IL-17 GES corresponded to tumor infiltration with T cells and neutrophils. Accordingly, high neutrophil infiltration correlated with clinical response specifically to dual ICI, and tumor-associated neutrophils also showed strong IL-17–T_H_17 pathway activity and T cell activation capacity. Both the blockade of IL-17A and the depletion of neutrophils impaired dual-ICI response and decreased T cell activation. Finally, high IL-17A levels in the blood of patients with melanoma indicated a higher global T_H_17 cytokine profile preceding clinical response to dual ICI but not to anti-PD-1 monotherapy, suggesting a future role as a biomarker for patient stratification.

## Abstract (web summary)

Roesch and colleagues use clinical datasets and mouse models of *BRAF*-mutant melanoma to reveal a role for IL-17A in positive responses to anti-PD-1 and anti-CTLA-4 therapy, which they also link to infiltrating neutrophils.

## Main

Treatment with immune checkpoint inhibition (ICI) has substantially improved survival of patients with metastatic melanoma (MM). Unfortunately, not all patients benefit to the same extent, as the majority relapses or experiences severe immune-related adverse events (irAEs). Still, there is a lack of feasible biomarkers and mechanistic understanding for risk stratification of patients with melanoma before ICI therapy. For example, in the CheckMate 067 study, treatment with the anti-PD-1 antibody nivolumab combined with the anti-CTLA-4 antibody ipilimumab (‘dual ICI’) showed a higher 6.5-year overall survival (OS) rate at 49% as opposed to 42% and 23% in the nivolumab and ipilimumab arms, respectively. The frequency of grade 3 and 4 toxicities was 59% with nivolumab plus ipilimumab, significantly higher than with nivolumab or ipilimumab alone (24% and 28%)^1^.

However, one unexpected observation from this study was that patients with *BRAF*^V600^ mutations in the nivolumab plus ipilimumab group survived longer than *BRAF*-wild-type (WT) patients (6.5-year OS rate of 57% versus 46%, median progression-free survival (PFS) of 16.8 versus 11.2 months). Interestingly, in the nivolumab and ipilimumab monotherapy arms, there were no or only small survival differences when stratified according to *BRAF* mutations^1,2^. Accordingly, also in the IMMUNED trial, patients with *BRAF*^V600^ mutations benefited from nivolumab plus ipilimumab more than *BRAF*-WT patients (hazard ratio (HR) for risk of recurrence or death, 0.11 versus 0.44, *P* = 0.019)^3^. Thus, unraveling *BRAF*-associated immunological pathways may lead to better understanding of the biologic mediators of therapeutic response to dual ICI and could provide a rationale to stratify patient treatment upfront.

The IL-17 family includes six structurally relevant members (IL-17A–IL-17F) and is a pro-inflammatory cytokine produced by a subset of CD4^+^ T cells, primarily type 17 helper T (T_H_17) cells^4,5^, CD8^+^ T cells and various innate immune cell types^6^. Compelling evidence suggests that IL-17 has an essential role in a multitude of autoimmune diseases and inflammation^7^. While several reports suggest that particularly inflamed tumors respond better to ICI^8^, it is controversial whether T_H_17–IL-17 inflammation could have an anti-tumor effect in melanoma, particularly during combined anti-PD-1 and anti-CTLA-4 therapy.

In this Article, it suggests that melanomas with pre-existent IL-17 signaling at therapy baseline benefit more from dual-ICI therapy. IL-17 signaling creates a favorable tumor microenvironment with increased immune infiltration, including neutrophils, and fosters T cell activation in preclinical melanoma mouse models and across different melanoma patient cohorts.

## Results

### The IL-17 pathway predicts clinical response to dual ICI

**Fig. 1:**
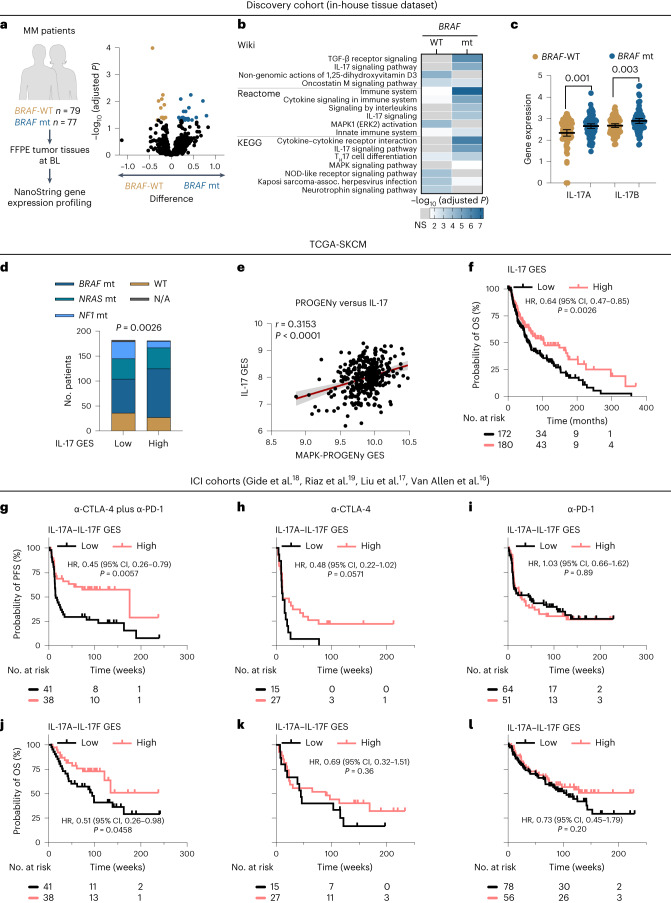
IL-17 pathway genes are associated with improved response to dual ICI. **a**, Left, schematic representation of the discovery cohort. Right, volcano plot showing the difference in *BRAF*-WT (*n* = 79 V600-negative samples)- and *BRAF*-mutant (*n* = 77 V600-positive samples)-associated gene expression (log_2_ (values)) and *q* values (−log_10_ (adjusted *P* values) from multiple unpaired *t*-tests with Benjamini, Krieger and Yekutieli test correction) in the discovery cohort. Each dot represents a gene; significant differentially expressed genes (*q* < 0.05) are shown in a color-coded manner. **b**, Heatmap showing enrichment scores (−log_10_ (adjusted *P* values), Benjamini–Hochberg-corrected FDR) of functional pathways in Wiki, Reactome and KEGG pathway databases. **c**, Scatter dot plots showing gene expression of *IL17A* and *IL17B* (*n* = 79 *BRAF*-WT, *n* = 77 *BRAF*-mutant tumors). Dots represent biologically independent patient samples. Mean ± 95% CIs are plotted; *P* values are from the unpaired *t*-test. **d**, Stacked bar plot showing the number of patients according to IL-17 signaling GES (according to KEGG hsa04657; cut point at median) and mutational subgroups in the TCGA-SKCM cohort (*n* = 363 tumor tissues). The *P* value is from the *χ*^2^ test. **e**, Scatterplot showing the correlation between IL-17 and the PROGENy MAPK activation GES in the TCGA-SKCM cohort (*n* = 363 tumor tissues). The line is from linear regression ±95% CI bands. **f**, Kaplan–Meier plot for OS according to the IL-17 signaling GES (KEGG hsa04657) in the TCGA-SKCM cohort. **g**–**l**, Kaplan–Meier plots for PFS (**g**–**i**) and OS (**j**–**l**) according to the IL-17 family GES (‘IL-17A–IL-17F GES’, IL-17 family cytokines containing the six structurally related cytokines) in patients treated with dual-ICI (**g**,**j**), mono anti-CTLA-4 (**h**,**k**) and mono anti-PD-1 (**i**,**l**) therapy. **g**–**l**, HR and 95% CIs are reported for high-expression groups. *P* values were calculated with the log-rank test. Categorization into ‘high’ versus ‘low’ was done according to an optimal cut point. All *P* values are two tailed. mt, mutant; FFPE, formalin fixed, paraffin embedded; BL, baseline; NS, not significant; NOD, nucleotide-binding oligomerization domain; assoc., associated; α, anti; N/A, not available. Source data

**Extended Data Fig. 1:**
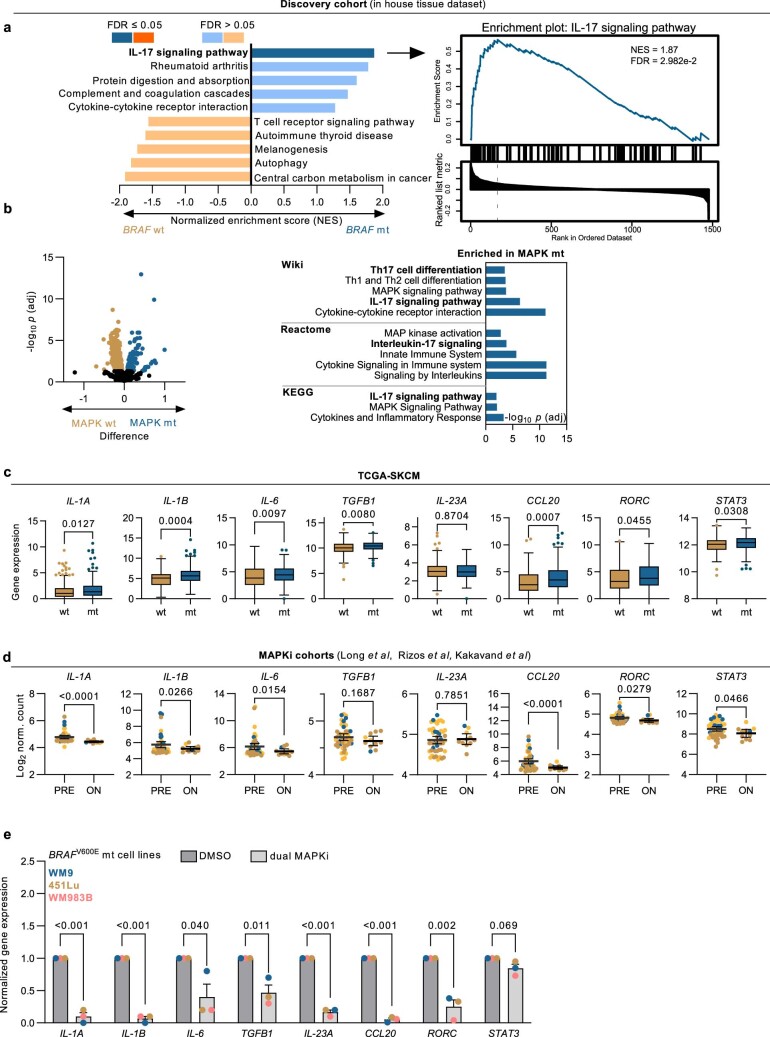
The association between the IL-17A signaling and MAPK pathways. (a) Gene set enrichment analysis in the discovery cohort showing (left) the normalized enrichment scores in pathways according to significance level and the corresponding enrichment plot for IL-17 signaling pathway (right). (b) Volcano plot showing the difference in MAPK wt (n = 36 triple wt tumors) and MAPK mt (n = 120 tumors with BRAF/NRAS hotspot and NF1 mutated tumors) associated gene expression (log2 values) and q-values (-log10 adjusted p-values from multiple unpaired t-test with Benjamini, Krieger and Yekutieli test correction) in the discovery cohort. Each dot represents a gene; significant DEGs (q < 0.05) are shown in a color-coded manner (left). Bar plot showing the enrichment scores (-log10 adjusted p-values, Benjamini–Hochberg corrected FDR) of functional pathways as defined by the Wiki, Reactome, and KEGG pathway databases (right). (c) Box and whiskers plots for gene expression of Th17/IL-17-inducing genes in the TCGA-SKCM cohort grouped according to BRAF status (n = 197 wt and n = 166 mt biologically independent tumors). Boxplot show the median (line) and interquartile ranges (Tukey whiskers that extend to 1.5 × IQR); p-values represent Mann-Whitney U test. (d) Scatter dot plots for gene expression of Th17/IL-17-inducing genes in the MAPKi dataset (Long et al, Rizos et al, and Kakavand et al datasets: GSE61992, GSE50509, GSE99898 series combined) grouped according to sample collection time point (PRE: before, ON: during therapy). Dots represent biologically independent tissues (n = 47 ON, n = 11 PRE) and are color-coded according to dataset; shown is mean ± 95% CI; p – values are from unpaired t-test. (e) qPCR analysis of BRAF mt (WM9, WM983B, 451Lu) melanoma cells treated with 1 nM dabrafenib/0.2 nM trametinib vs. DMSO for 7 days. Bar plot shows mean ± SEM where single dots represent biologically independent cell lines; p-values are from unpaired t-test. Shown is one representative out of three independently performed experiments. All p-values are two-tailed. mt: mutant, wt: wild-type, GSEA: gene set enrichment analysis, FDR: false discovery rate, NES: normalized enrichment score, TCGA: The Cancer Genome Atlas, SKCM: Skin cutaneous melanoma, MAPKi: mitogen-activated protein kinase inhibitor, IQR: interquartile range. Source data

To find a molecular rationale for ICI therapy prediction in patients with melanoma based on the observed difference in response to dual ICI between *BRAF*-mutant and *BRAF*-WT melanomas, we performed gene expression profiling of treatment-naive archived tumor samples (discovery set: *n* = 77 *BRAF*-mutant (V600 hotspot-positive), *n* = 79 *BRAF*-WT melanomas; Fig. 1a, left). To reveal GES in therapeutically relevant immune and resistance pathways, we applied NanoString technology due to its analytical robustness with optimized detection of low-expression RNA targets in formalin-fixed paraffin-embedded material. The baseline clinical characteristics of the discovery cohort and details on the NanoString gene panels have been recently described^9^. Differential gene expression analysis revealed diverging transcriptional landscapes between *BRAF*-mutant and *BRAF*-WT tumors. There were 21 transcripts significantly upregulated in *BRAF*-mutant tumors with enrichment for cytokine- and chemokine-encoding genes (Fig. 1a,b and Supplementary Table 1). In particular, we found transcriptional signatures indicative of interleukin signaling, especially IL-17, and associated T_H_17 cell differentiation pathways being over-represented in *BRAF*-mutant tumors based on pathway enrichment and gene correlation analyses (Fig. 1b,c). In addition, gene set enrichment analysis confirmed IL-17 GES upregulation in *BRAF*-mutant tumors (Extended Data Fig. 1a).

As it has been described that IL-17 signaling requires mitogen-activated protein kinase (MAPK) activation^10,11^, we expanded our analyses to common oncogenic MAPK mutations beyond *BRAF*^V600^. Both IL-17 and T_H_17 cell differentiation GES were among the most significantly over-represented pathways in MAPK-mutated (*n* = 77 *BRAF* hotspot-mutant, *n* = 42 *NRAS* hotspot-mutant, *n* = 1 *NF1*-mutant) melanomas compared to triple-WT melanomas (*n* = 36) (Extended Data Fig. 1b). To further validate the link between IL-17 signaling GES (defined according to Kyoto Encyclopedia of Genes and Genomes (KEGG) hsa04657) and the MAPK pathway, we analyzed data from the largest available melanoma dataset from the Cancer Genome Atlas (TCGA) Skin Cutaneous Melanoma (TCGA-SKCM) cohort and found a significant association between IL-17 GES and the MAPK mutational state (Fig. 1d). Furthermore, this association was also significant when we correlated IL-17 GES with the transcriptional oncogenic activation signature of the MAPK pathway (MAPK-Pathway Responsive Genes (PROGENy)^12^; Fig. 1e).

The MAPK pathway plays a role in cellular survival and proliferation, but it is also involved in the production and expression of pro-inflammatory cytokines. Therefore, we correlated the oncogenic activation of the MAPK pathway in melanoma cells with specific cytokines known to regulate IL-17 induction. We found that several IL-17-inducing genes were expressed at higher levels in *BRAF*-mutant than in *BRAF*-WT tumors in the SKCM cohort and that their expression was significantly decreased in MAPK inhibitor (MAPKi)-treated melanoma tissue biopsies^13–15^ (Extended Data Fig. 1c,d). To further confirm the regulatory axis between MAPK activation and IL-17 regulators, we demonstrated, by pharmacologic manipulation in vitro, that IL-17-inducing genes can be expressed by *BRAF*-mutant melanoma cells themselves, and dual MAPKi (dabrafenib plus trametinib) leads to decreased transcription of IL-17-regulatory genes (Extended Data Fig. 1e).

To investigate a potentially relevant prognostic value of baseline IL-17 GES in melanoma tissues that is universal and not necessarily dependent only on MAPK signaling, we explored the association between OS and IL-17 signaling in the TCGA-SKCM dataset that mainly consists of untreated melanoma tumors. Indeed, high IL-17 GES was significantly associated with improved OS (HR, 0.64; 95% confidence interval (CI), 0.47–0.85; *P* = 0.0026; Fig. 1f). Next, we analyzed four different RNA-seq datasets from ICI-treated patient cohorts with MM of various genotypes (combined cohorts of anti-CTLA-4, anti-PD-1 or anti-CTLA-4 and anti-PD-1 therapy; Van Allen et al., Liu et al., Riaz et al. and Gide et al.; exact patient numbers are provided in the Methods)^16–19^. Intriguingly, high expression of core IL-17 signaling genes (‘IL-17A–IL-17F GES’, IL-17 family cytokines containing the six structurally related cytokines) predicted longer PFS in dual-ICI-treated patients (HR, 0.45; 95% CI, 0.26–0.79; *P* = 0.0057), while it did not correlate with treatment response to anti-PD-1 or anti-CTLA-4 monotherapy (Fig. 1g–i). High IL-17 signaling was also associated with longer OS in dual ICI (HR, 0.51; 95% CI, 0.26–0.98; *P* = 0.0458) but not in ICI monotherapies (Fig. 1j–l).

Overall, these results suggest co-regulation of the IL-17 and the MAPK pathway, particularly in *BRAF*-mutant melanomas in which there is strong MAPK activation. However, IL-17 pathway activity is probably not restricted to (known) oncogenic MAPK activators and may instead be a universal predictor of response to ICI.

### IL-17A is crucial for response to dual ICI in mouse melanoma

**Fig. 2:**
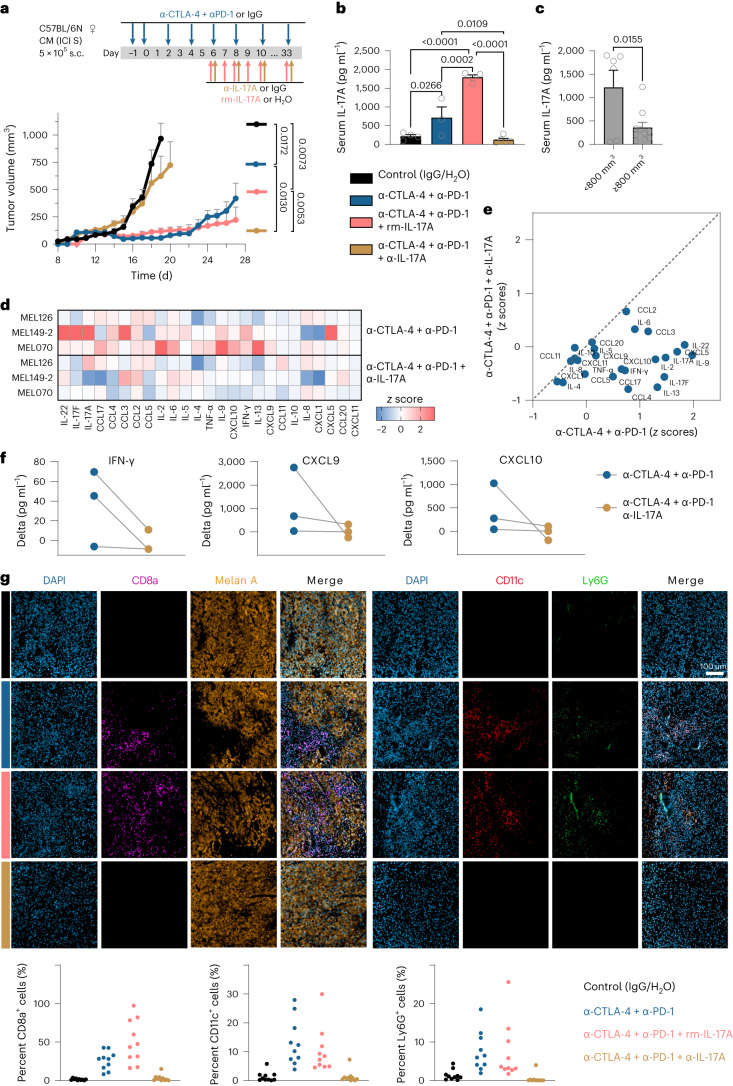
IL-17A supports anti-tumor effects of dual ICI. **a**, Tumor growth kinetics of transplanted CM (*BRAF*-WT ICI-sensitive) melanoma tumors treated with immunoglobulin G (IgG) or H_2_O (control, *n* = 6), anti-CTLA-4 + anti-PD-1 (*n* = 6), anti-CTLA-4 + anti-PD-1 + rm-IL-17A (*n* = 6) and anti-CTLA-4 + anti-PD-1 + α-IL-17A (*n* = 6) antibodies according to the depicted treatment schedule. Data points show mean + s.e.m. until the day when the first mice were eliminated from each group; *P* values are from one-way ANOVA with Tukey’s multiple-comparison test. **b**, Serum IL-17A levels from the endpoint measurement (day 33) by ELISA. Shown are mean + s.e.m. of *n* = 3–5 biologically independent samples per group; *P* values are from one-way ANOVA with Holm–Sidak’s multiple-comparison test. **c**, Corresponding serum IL-17A levels in mice grouped according to final tumor volume (*n* = 6 (<800 mm^3^) versus *n* = 10 (≥800 mm^3^) biologically independent samples). The bar plot shows mean + s.e.m., and the *P* value is from an unpaired *t*-test. **d**,**e**, Heatmap (**d**) and corresponding *x*–*y* plot (**e**) with *z* scores representing the normalized delta (stimulated − unstimulated condition) values of soluble mediators secreted by PDTFs from *n* = 3 human melanoma tumors treated ex vivo with anti-CTLA-4 + anti-PD-1 or with anti-CTLA-4 + anti-PD-1 + α-IL-17A antibodies. **f**, Delta values of IFN-γ, CXCL10 and CXCL9 secreted by PDTFs upon either anti-CTLA-4 + anti-PD-1 or anti-CTLA-4 + anti-PD-1 + α-IL-17A ex vivo treatment. **g**, Representative immunostaining images of CM tumors (day 9) showing melanoma (melan A) and immune cell markers (CD8a, CD11c, Ly6G). Scatter dot plots show the relative contribution of immune cells (*n* = 5 random fields per whole-tumor area, normalized to 4,6-diamidino-2-phenylindole (DAPI) values; *n* = 2 biologically independent tumors per group). All *P* values are two tailed. S, sensitive; s.c., subcutaneous; ♀, female; MEL, melanoma; CCL, C–C motif chemokine ligand; TNF, tumor necrosis factor. Source data

**Extended Data Fig. 2:**
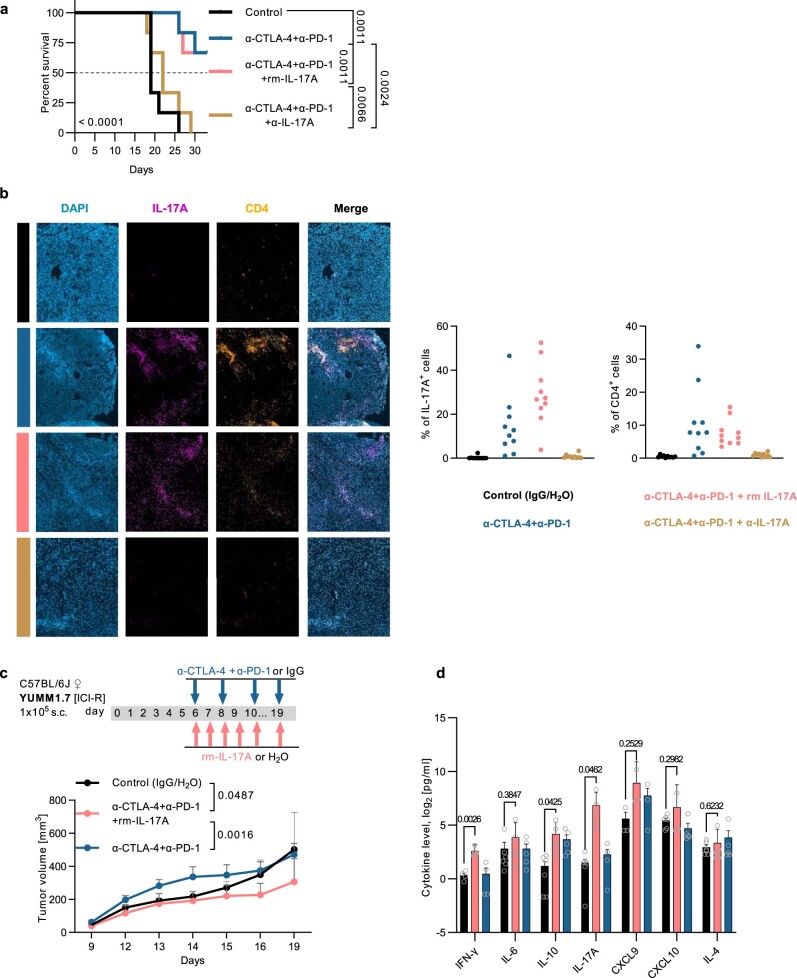
IL-17A supports anti-tumor effects of dual ICI in mouse melanoma. (a) Kaplan Meier plot related to Fig. 2a, showing survival of mice. p-values are from log rank test. (b) Extended immunostaining panel related to Fig. 2e showing IL-17A and CD4 positivity in the CM (BRAF wt, ICI-sensitive) mouse model. Corresponding scatter dot plots of immunostaining quantification (n = 5 random fields/whole tumor area normalized to DAPI; n = 2 biologically independent tumors/group). (c) Tumor growth kinetics of YUMM1.7 (BRAF mt, ICI-resistant) melanoma treated with IgG/H2O (control, n = 5), α-CTLA-4 + α-PD-1 (n = 4), α-CTLA-4 + α-PD-1 plus rm-IL-17A (n = 4) according to treatment schedule as depicted. Data points show mean + SEM, and p-values are from 1-way ANOVA with Holm-Sidak’s multiple comparisons test. (d) Corresponding cytokine and chemokine concentrations as quantified by a multiplex cytokine array in endpoint serum samples (day 19). Bar plot shows n = 3 to 6 biologically independent samples/group. Data points show mean + SEM, and p-values are from unpaired t-test. All p-values are two-tailed. Source data

To study the effect of the systemic IL-17A level on the anti-tumor efficacy of ICI therapy in vivo, we used two syngeneic melanoma transplantation models with distinct genotypes and response profiles to experimentally administered anti-CTLA-4 and anti-PD-1 antibodies^20,21^. First, we examined the effects of an IL-17A-neutralizing antibody (α-IL-17A) and recombinant mouse IL-17A (rm-IL-17A) on tumor growth kinetics in the ICI-sensitive MT/*ret*-derived primary cutaneous melanoma (CM) mouse model (human *ret* transgene, *BRAF*-WT^20^). As expected, dual ICI significantly slowed down CM tumor growth compared to controls (*P* = 0.0172). Treatment with dual ICI in combination with rm-IL-17A also decreased tumor growth (*P* = 0.0073 versus controls), whereas the addition of α-IL-17A strongly blocked the anti-tumor effect of dual ICI (*P* = 0.0130 versus dual ICI; Fig. 2a) and significantly shortened survival (Extended Data Fig. 2a). Endpoint analysis of serum IL-17A levels confirmed that the addition of α-IL-17A resulted in significantly less serum IL-17A than levels from dual-ICI-treated mice (*P* = 0.0109; Fig. 2b). Furthermore, we found a negative correlation between tumor size and serum IL-17A levels, with especially large aggressive tumors (≥800 mm^3^) having significantly lower IL-17A concentrations (*P* = 0.0155; Fig. 2c).

To understand whether IL-17A is also a relevant contributor to CTLA-4 and PD-1 blockade in human melanomas, we used an ex vivo patient-derived tumor fragment (PDTF) model, which has recently demonstrated high predictive capacity for ICI^22,23^. PDTFs from three ICI-responsive patient melanomas were treated with dual ICI in the absence or presence of α-IL-17A. In line with the effects observed in mouse models, α-IL-17A decreased immune activation upon dual ICI and particularly abrogated IFN-γ-induced responses, which is known as a critical driver of clinical response to ICI^24^ (Fig. 2d–f).

Next, we characterized the tumor microenvironment to unravel the landscape of IL-17-mediated early immune cell infiltration. We set up a short-term treatment regimen in the CM model (using the same drug doses) and performed multiplex immunofluorescence staining. Overall, tumors treated with dual ICI alone or in combination with rm-IL-17A had higher immune cell infiltration than the control. In particular, CD8^+^ T cells that are the main effectors of therapeutic ICI^25^ were increased in tumors treated with dual ICI alone or in combination with rm-IL-17A. Furthermore, CD4^+^ cells, IL-17A^+^ cells, CD11c^+^ cells and Ly6G^+^ neutrophils that are potential downstream effectors of IL-17 functions were also significantly enriched in tumors treated with dual ICI alone or in combination with rm-IL-17A, whereas the addition of α-IL-17A counteracted the effect of dual ICI and prevented immune cell infiltration (Fig. 2g and Extended Data Fig. 2b).

Second, we asked whether IL-17 could improve ICI responsiveness also in an intrinsically resistant tumor scenario and applied the YUMM1.7 mouse model, which was reported to lack response to ICI (*Pten*^del^, *Cdkn2a*^del^, *BRAT*^V600E^-mutant melanoma^21^). As expected, YUMM1.7 tumors treated with dual ICI showed no response, and mice developed tumors similar to the control (*P* > 0.05 versus control). However, addition of rm-IL-17A significantly slowed down tumor growth (*P* = 0.0487 versus control, *P* = 0.0016 versus dual ICI; Extended Data Fig. 2c). Endpoint analysis of serum samples revealed that addition of rm-IL-17A to dual ICI resulted in increased production of the T cell chemokines IFN-γ, CXCL9 and CXCL10, which have been shown to play a role in ICI response and CD8^+^ T cell recruitment^26^ (Extended Data Fig. 2d). Together, these findings indicated that increased IL-17 signaling contributes to better response in dual ICI.

### The IL-17-associated cellular microenvironment in dual ICI

**Fig. 3:**
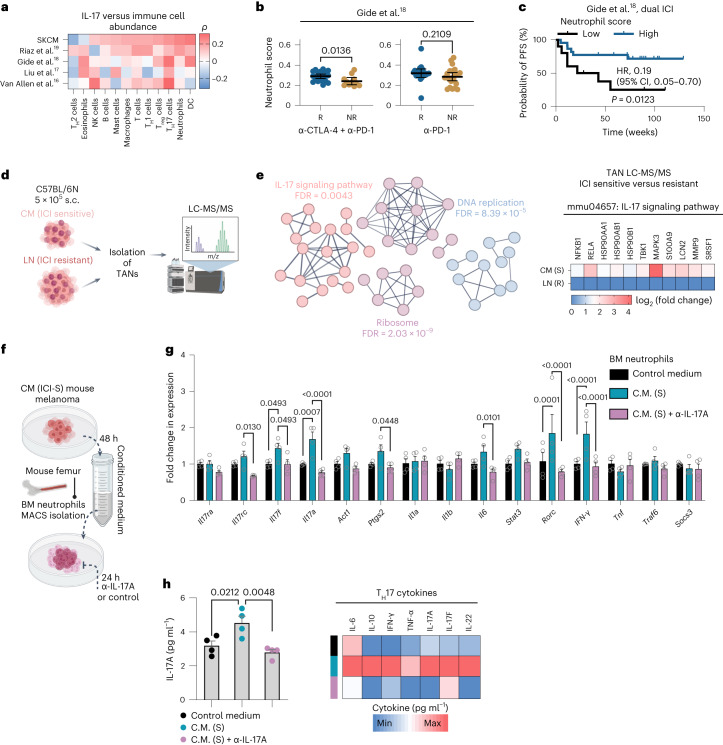
The IL-17 signaling-associated cellular microenvironment in melanomas treated with ICI. **a**, Heatmap showing Spearman’s correlation between immune cell types (following the Bindea et al.^51^ algorithm) and IL-17A–IL-17F GES in tumor samples in bulk RNA-seq cohorts. **b**, Scatter dot plots showing estimated neutrophil cell enrichment in baseline tissue samples of therapy responders (*n* = 21) versus non-responders (*n* = 11) treated with dual ICI (left) and therapy responders (*n* = 19) versus non-responders (*n* = 22) treated with anti-PD-1 monotherapy (right) in the Gide et al.^18^ dataset. *P* values are from unpaired *t*-tests, and mean ± 95% CIs are plotted. Each dot represents a biologically independent sample. **c**, Kaplan–Meier plot for PFS according to baseline neutrophil cell enrichment levels (*n* = 10, ‘low’; *n* = 22, ‘high’, according to an optimal cut point) in the dual-ICI group of Gide et al.^18^. HRs and 95% CIs are reported for high-expression groups, and the *P* value is from the log-rank test. **d**,**e**, Schematic workflow (**d**) for LC-MS/MS analysis (**e**). LC/MS/MS icon created by BioRender.com. Search Tool for the Retrieval of Interacting Genes/Proteins (STRING) analysis showing the top significantly enriched cellular pathways in TANs derived from CM tumors. The corresponding heatmap shows fold change in protein expression of IL-17 signaling pathway (according to KEGG mmu04657) components. **f**, Schematic workflow for naive BM neutrophil isolation and in vitro analysis. **g**, Quantitative PCR (qPCR) analysis of key IL-17 signaling components in untreated versus α-IL-17A-treated (5 µg ml^−1^) naive BM neutrophils cultured for 24 h in conditioned medium derived from CM mouse melanoma. Regular growth medium (no exposure to tumor cells) was used as a control. Bar plots represent mean + s.e.m. from *n* = 4 biologically independent samples; *P* values are from two-way ANOVA with Holm–Sidak’s multiple-comparison test. Shown is one representative of three independently performed experiments. **h**, Corresponding IL-17A (left, bar plot) and T_H_17 cytokine levels (right, heatmap) in the supernatants of BM neutrophils from **g**. The bar plot shows mean + s.e.m.; dots represent individual biological replicates. *P* values are from one-way ANOVA with Sidak’s multiple-comparison test. C.M., conditioned medium; R, responder; NR, non-responder; DC, dendritic cell; MACS, magnetic-activated cell sorting; max, maximum; min, minimum; T_H_, helper T cell; T_reg_, regulatory T cell. Source data

In silico analysis of different bulk RNA-seq datasets showed that high IL-17 expression is positively correlated with high presence of T_H_17 cells and T cells, dendritic cells, mast cells and neutrophils (Fig. 3a). Notably, IL-17-associated elevation of T_H_17 cells, dendritic cells and neutrophils is already present in untreated tumors (TCGA-SKCM data), suggesting that subgroups of melanomas harbor a pre-existent immune composition that may determine susceptibility to dual ICI upfront to therapy.

IL-17 is known to activate innate immune mechanisms by inducing expression of pro-inflammatory cytokines and recruitment of neutrophils^4^. Accordingly, we found that neutrophil gene signatures are significantly enriched in baseline tumors of dual-ICI responders (*P* = 0.0136) but not in mono anti-PD-1 responders (*P* = 0.2109, Gide et al. dataset^18^; Fig. 3b). Moreover, high neutrophil abundance at baseline correlated with longer PFS (HR, 0.19; 95% CI, 0.05–0.70; *P* = 0.0123) in the dual-ICI cohort (Fig. 3c).

**Extended Data Fig. 3:**
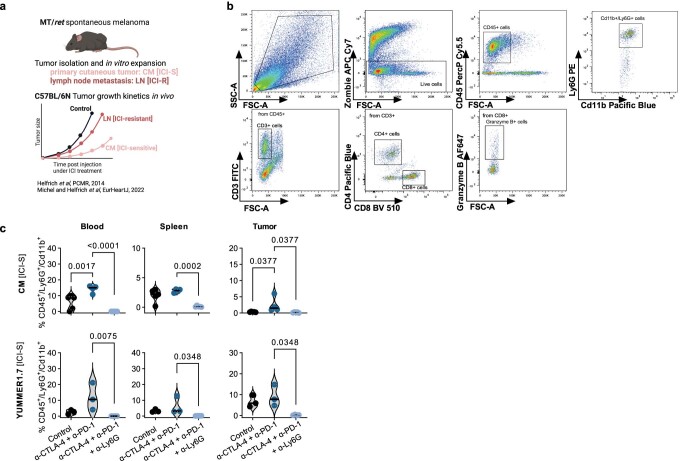
Experimental details of neutrophil in vivo experiments. (a) Schematic workflow for LC-MS/MS analysis. (b) Flow cytometry gating strategy. (c) Violin plots show the distribution of Ly6G+ neutrophils in blood, spleen, and tumor tissues of mice from Fig. 4a,b. p-values are from 1-way ANOVA with Holm-Sidak’s multiple comparisons test (top panels, CM model) and from Kruskal-Wallis test with Dunn’s multiple comparisons test (bottom panels, YUMMER1.7 model). All p-values are two-tailed. LC-MS/MS: liquid chromatography-mass spectrometry/mass spectrometry. Source data

To experimentally validate the role of neutrophils in response to ICI, we injected C57BL/6N mice either with ICI-sensitive MT/*ret* CM cells or the ICI-resistant MT/*ret* LN subline (derived from a single resistant lymph node^20^; Extended Data Fig. 3a) and expanded the tumors to a size of ~250 mm^3^. We then isolated tumor-associated neutrophils (TANs) from both models and performed liquid chromatography-mass spectrometry/mass spectrometry (LC-MS/MS) (Fig. 3d). We analyzed the differentially expressed proteins and enriched functional pathways between ICI-sensitive and ICI-resistant TANs and found a significantly higher expression of proteins belonging to DNA replication, ribosome and the IL-17 signaling pathway in ICI-sensitive TANs (Fig. 3e). Next, we isolated naive bone marrow (BM) neutrophils from C57BL/6N mice and cultured them in conditioned medium derived from ICI-sensitive CM melanoma cells with or without α-IL-17A for 24 h (Fig. 3f). We confirmed that several IL-17 signaling genes were expressed at a significantly higher level in BM neutrophils stimulated with conditioned medium from intrinsically ICI-sensitive mouse melanoma and that this was abrogated by concurrent α-IL-17A treatment (Fig. 3g). Similar results were seen for IL-17A and other T_H_17 cytokine levels in corresponding cell culture supernatants (Fig. 3h).

### The IL-17-associated role of neutrophils in dual ICI

**Fig. 4:**
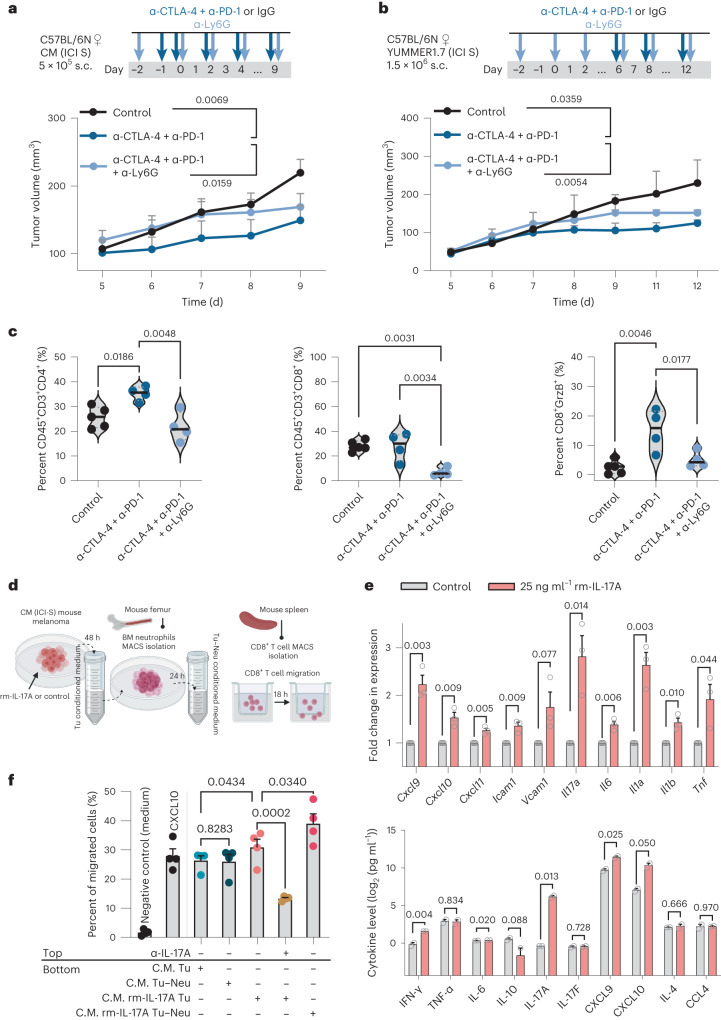
The IL-17-associated presence of neutrophils plays a role in the anti-tumor response to dual ICI. Tumor growth kinetics of CM (*BRAF*-WT ICI-sensitive) (**a**) and YUMMER1.7 (*BRAF*-mutant ICI-sensitive) (**b**) tumors treated with IgG or H_2_O (control, CM, *n* = 5; YUMMER1.7, *n* = 4), anti-CTLA-4 + anti-PD-1 antibodies (CM, *n* = 4; YUMMER1.7, *n* = 4) or anti-CTLA-4 + anti-PD-1 + anti-Ly6G antibodies (CM, *n* = 5; YUMMER1.7, *n* = 4) according to the depicted treatment schedule. Data points show mean + s.e.m.; *P* values are from one-way ANOVA with Holm–Sidak’s multiple-comparison test. **c**, Violin plots showing tumor immune cell frequencies by flow cytometry from **a** (CM model). *P* values are from one-way ANOVA with Holm–Sidak’s multiple-comparison test. **d**, Schematic workflow for in vitro culture, isolation of BM neutrophils and splenic CD8^+^ T cells followed by the migration assay. **e**, Top, qPCR analysis of transcripts encoding T cell chemokines, adhesion molecules and T_H_17 signaling components in the control or rm-IL-17A-treated CM mouse cell line. Bar plots represent mean + s.e.m. from *n* = 3 biological replicates; *P* values are from the unpaired *t*-test. Shown is one representative of two independently performed experiments. Bottom, corresponding cytokine and chemokine levels in cell culture supernatants of CM cells treated with rm-IL-17A. Data points show mean + s.e.m. from *n* = 2 biologically independent samples; *P* values are from the unpaired *t*-test. **f**, Bar plot showing the percentage of migrated CD8^+^ T cells in the Boyden chamber assay. The different medium conditions used as chemoattractant in the bottom chamber are depicted below the horizontal line. Serum-free medium was used as the negative control, and recombinant mouse CXCL10 (200 ng ml^−1^) was used as the positive control. α-IL-17A (5 µg ml^−1^) was added to the top chamber (depicted above the horizontal line) as indicated. Individual data points represent *n* = 3–4 biological replicates per group; *P* values are from one-way ANOVA with Sidak’s multiple-comparison test. Shown is one representative of three independently performed experiments. All *P* values are two tailed. Tu, tumor; Neu, neutrophil; Grz, granzyme. Source data

Next, we applied an anti-Ly6G antibody that specifically depletes neutrophils^27^ and combined it with dual ICI in two independent ICI-sensitive transplantation models (CM and YUMMER1.7). To avoid regeneration and expansion of BM neutrophils, we monitored short-term tumor growth kinetics. We verified that neutrophil depletion with the anti-Ly6G antibody technically worked in both models, evident by the reduced frequency of CD45^+^CD11b^+^Ly6G^+^ cells in blood, spleen and tumor samples collected at day 9 and day 12 (CM and YUMMER1.7 models, respectively; Extended Data Fig. 3c). Addition of the anti-Ly6G antibody to anti-CTLA-4 and anti-PD-1 antibodies significantly accelerated tumor growth and weakened the dual-ICI response in both models (Fig. 4a,b). Furthermore, flow cytometry analysis of intratumoral immune cell frequencies indicated that the increase in CD4^+^ and CD8^+^ T cells in dual-ICI-treated tumors was counteracted by the anti-Ly6G antibody and that the frequency of intratumoral cytotoxic CD8^+^ T cells (CD8^+^granzyme B^+^ cell fraction) was significantly reduced in a neutrophil-lacking tumor microenvironment (Fig. 4c).

Because these results indicated possible crosstalk between TANs and cytotoxic mediators of the ICI response, we set up in vitro experiments to study the migration capacity of murine CD8^+^ T cells. We first generated conditioned medium from untreated and rm-IL-17A-treated ICI-sensitive CM mouse melanoma cells (tumor conditioned medium). In subsequent steps, the tumor cell-derived conditioned medium was used to culture naive BM neutrophils. After 24 h of culturing, the conditioned medium from the neutrophils was also collected (tumor neutrophil conditioned medium) and used as chemoattractant in CD8^+^ T cell migration Boyden chamber assays (Fig. 4d). Treatment with rm-IL-17A led to increased mRNA expression of key T cell chemokines such as CXCL9–CXCL11, adhesion molecules ICAM1 and VCAM1 and IL-17-dependent cytokines in melanoma cells. The corresponding cell culture supernatants also showed a significant increase in T cell chemokines and T_H_17 cytokines (Fig. 4e). Consequently, conditioned medium from rm-IL-17A-treated melanoma cells attracted more CD8^+^ T cells than conditioned medium from untreated cells (*P* = 0.0434). Importantly, migration was significantly reduced by concurrent α-IL-17A treatment of CD8^+^ T cells (*P* = 0.0002; Fig. 4f). Finally, CD8^+^ T cell migration was increased when we used conditioned medium from tumor neutrophils treated with rm-IL-17A as compared to conditioned medium from rm-IL-17A-treated melanoma cells alone (*P* = 0.0340), while CD8^+^ T cell migration remained at a similar level when conditioned media from untreated melanoma cells versus untreated tumor neutrophils were used (*P* = 0.8283; Fig. 4f).

Overall, these results suggest that, in dual-ICI-sensitive melanoma, a tumor baseline scenario characterized by high IL-17 pathway activity and neutrophil accumulation positively stimulates T cell migration and tumor elimination.

### IL-17A and T_H_17 cytokines predict the response to dual ICI

**Fig. 5:**
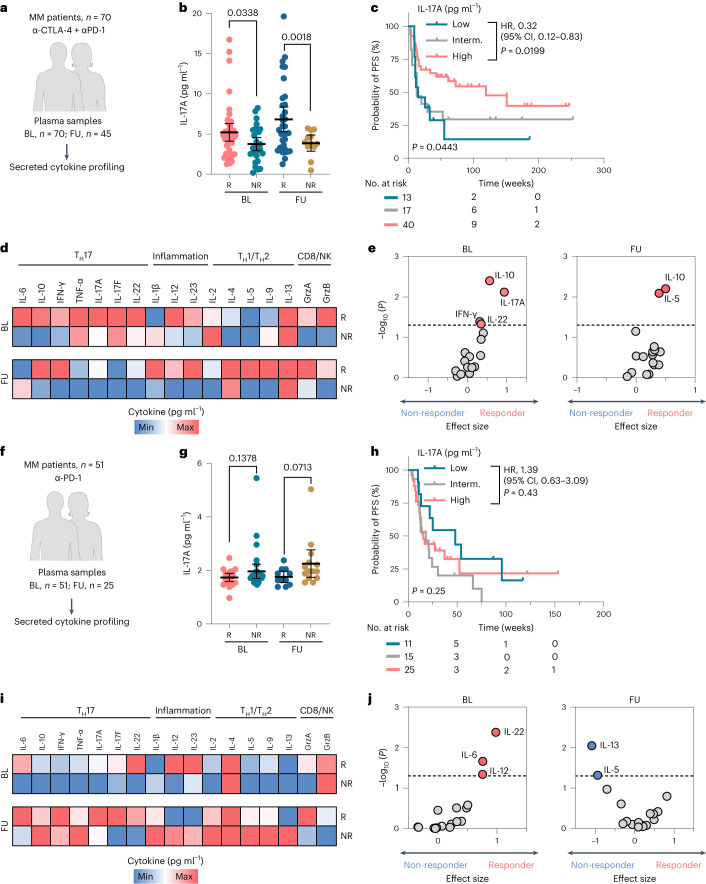
IL-17A–T_H_17 profiling for response prediction in ICI-treated patients with melanoma. **a**, Dual-ICI-treated melanoma patient cohort (first-line anti-CTLA-4 and anti-PD-1 therapy, *n* = 70). **b**, Plasma IL-17A levels as measured by ELISA in correlation to the best clinical response in samples collected at therapy baseline (*n* = 41 responders versus *n* = 29 non-responders) and at early follow-up (*n* = 33 responders versus *n* = 12 non-responders) visits. **c**, Kaplan–Meier plot for PFS according to the baseline IL-17A concentration. **d**, Heatmaps representing the median cytokine concentrations as quantified by multiplex cytokine array for responding versus non-responding patients. **e**, Corresponding volcano plot showing the effect size (Hedge’s *g*) and −log_10_ (*P* values) (Mann–Whitney *U*-test) for each cytokine for responding versus non-responding patients. **f**, Mono anti-PD-1-treated melanoma patient cohort (first-line anti-PD-1 therapy, *n* = 51). **g**, Plasma IL-17A levels as measured by ELISA in correlation to the best clinical response in samples collected at baseline (*n* = 19 responders versus *n* = 32 non-responders) and at early follow-up (*n* = 11 responders versus *n* = 14 non-responders) visits. **h**, Kaplan–Meier plot for PFS according to the baseline IL-17A concentration. **i**, Heatmaps representing median cytokine concentrations as quantified by multiplex cytokine array for responding versus non-responding patients. **j**, Corresponding volcano plot showing the effect size (Hedge’s *g*) and −log_10_ (*P* values) (Mann–Whitney *U*-test) for each cytokine for responding versus non-responding patients. *P* values are from the unpaired *t*-test with Welch’s correction, and mean ± 95% CIs are plotted in **b**,**g**. Each dot represents a biologically independent sample. Categorization into ‘high’ versus ‘low’ according to the X-tile-determined cut-point value was carried out separately within each dataset. HRs and 95% CIs are reported for ‘IL-17A high’, and *P* values are from the log-rank test in **c**,**h**. Significant predictors of response or non-response are shown above the dashed line (*P* < 0.05) in baseline or follow-up plasma samples in **e**,**j**. All *P* values are two tailed. FU, follow-up; responder, complete and partial responses; non-responder, progressive disease, mixed response; interm., intermediate. Source data

Our findings indicated thus far that IL-17 contributes to enhanced dual-ICI response and could serve as a therapy stratification biomarker. Therefore, we analyzed plasma samples of 121 patients with melanoma treated at the Essen Department of Dermatology with either first-line dual ICI (anti-CTLA-4 plus anti-PD-1 antibodies, *n* = 70) or with first-line anti-PD-1 monotherapy (*n* = 51) (Fig. 5a,f). Secreted IL-17A levels in samples collected at therapy baseline and also at early follow-up visits (median, week 9; range, 2–12 weeks) were significantly higher in therapy responders under dual-ICI treatment than in non-responders (*P* = 0.0338 at baseline, *P* = 0.0018 at follow-up; Fig. 5b). To test whether baseline IL-17A levels could be used as a biomarker for pre-therapeutic therapy stratification, we categorized patients according to their baseline IL-17A plasma concentrations. We applied the bioinformatic tool X-tile to achieve the optimal cut-point-based prognostication^28^. We found that dual-ICI-treated patients with a high baseline IL-17A concentration (≥3.76 pg ml^−1^) had longer PFS than patients with intermediate (2.30–3.75 pg ml^−1^; *P* = 0.0682; HR, 0.46) or low (≤2.29 pg ml^−1^; *P* = 0.0199; HR, 0.32) baseline IL-17A levels (Fig. 5c). To test whether elevated IL-17A is indicative of a global T_H_17 cytokine profile and phenotype induction, we applied a bead-based multiplex cytokine array including several known T_H_17, type 1 and 2 helper T cell, inflammatory and CD8^+^ T cell–natural killer (NK) (CD8/NK) activation-associated cytokines. Interestingly, dual-ICI therapy responders had higher T_H_17-associated cytokine (IL-10, IFN-γ, IL-17A and IL-22; *P* < 0.05) levels, particularly at baseline (Fig. 5d,e). While other inflammatory and CD8/NK cytokines were also elevated in baseline and follow-up samples from responders, they did not statistically stratify patients (Fig. 5d,e).

By contrast, response to anti-PD-1 monotherapy showed no statistically significant correlation with the plasma IL-17A level, although there was a non-significant trend for elevated IL-17 levels in non-responders (Fig. 5f–h). Analysis of additional cytokines in the mono anti-PD-1 cohort revealed differences between therapy responders versus non-responders to a lesser extent, with only baseline IL-6, IL-22 and IL-12 (*P* < 0.05) significantly stratifying patients according to response. Interestingly, and in contrast to dual-ICI responders, T_H_17 cytokines were higher in mono anti-PD-1 responders at follow-up but not in baseline plasma samples (statistically not significant, *P* = 0.5781; Fig. 5i,j).

**Fig. 6:**
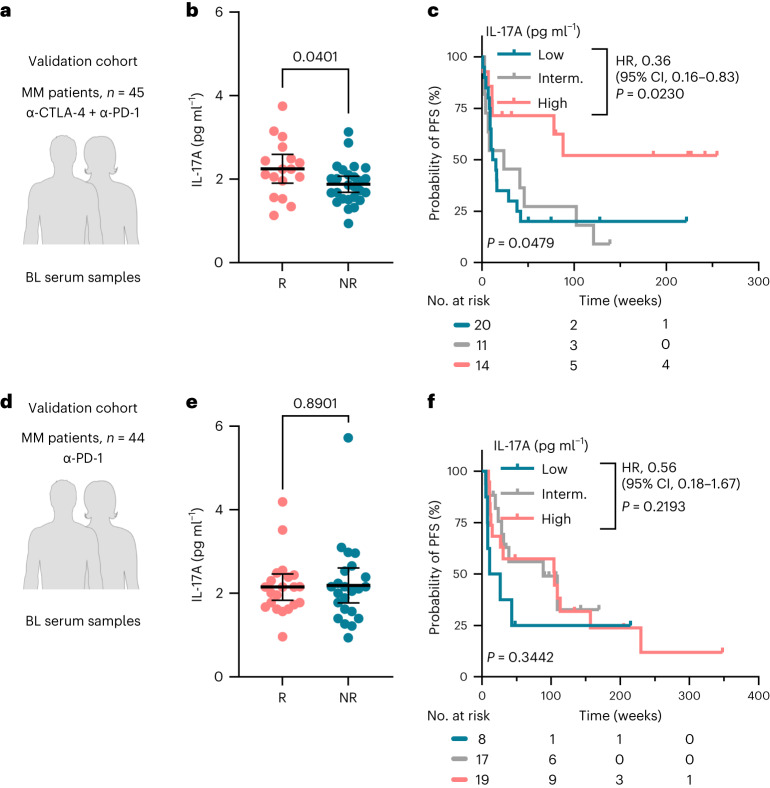
Validation cohort. **a**, Dual-ICI-treated melanoma validation cohort (anti-CTLA-4 and anti-PD-1 antibodies, *n* = 45). **b**, Serum IL-17A levels as measured by ELISA in correlation to the best clinical response (*n* = 17 responders versus *n* = 26 non-responders) in samples collected at therapy baseline. **c**, Kaplan–Meier plot for PFS according to the baseline IL-17A concentration. **d**, Mono anti-PD-1-treated melanoma validation cohort (anti-PD-1 therapy, *n* = 44). **e**, Serum IL-17A levels as measured by ELISA in correlation to the best clinical response (*n* = 21 responders versus *n* = 23 non-responders) in samples collected at therapy baseline. **f**, Kaplan–Meier plot for PFS according to the baseline IL-17A concentration. *P* values are from the unpaired *t*-test, and mean ± 95% CIs are plotted in **b**,**e**. Each dot represents a biologically independent sample. Categorization into ‘high’ versus ‘low’ according to the X-tile-determined cut-point value was carried out separately within each dataset. HRs and 95% CIs are reported for ‘IL-17A high’, and *P* values are from the log-rank test in **c**,**f**. All *P* values are two tailed. Responder, complete and partial response; non-responder, progressive disease, stable disease, mixed response. Source data

Finally, we validated these findings using a multi-center validation cohort. Baseline serum samples of 45 patients with melanoma treated with dual ICI (anti-CTLA-4 plus anti-PD-1 antibodies) and 44 patients with melanoma treated with anti-PD-1 monotherapy were independently collected across four different dermatology departments (Tübingen, Mannheim and Essen in Germany; St. Gallen in Switzerland; Fig. 6a,d). We confirmed that high baseline IL-17A levels were associated with dual-ICI response (*P* = 0.0401 responders versus non-responders; Fig. 6b) and longer PFS (*P* = 0.0230; HR, 0.36; Fig. 6c). By contrast, baseline IL-17A levels did not correlate with mono anti-PD-1 response (*P* > 0.05; Fig. 6e,f).

In conclusion, our data suggest that plasma IL-17 and T_H_17 cytokines may be a valuable baseline biomarker for response prediction and patient stratification in melanoma, specifically to predict a potential benefit of adding anti-CTLA-4 to anti-PD-1 antibodies upfront to therapy. For a deeper understanding of the dynamics of immune cytokine levels under ICI, for example, to switch treatment when resistance development is imminent, extended studies with systematic longitudinal sampling protocols are needed.

## Discussion

Following recent observations from clinical trials indicating that patients with *BRAF*-mutant melanoma in particular benefit from dual ICI^1–3^, we wondered whether we could derive a molecular rationale that prospectively leads to a more general biomarker concept for ICI therapy stratification. As a starting point, we analyzed transcriptional differences between *BRAF*-mutant versus *BRAF*-WT tumors in a NanoString discovery cohort specifically focusing on known immune and resistance signatures. We found IL-17 and related T_H_17 GES to be significantly enriched in *BRAF*-mutant tumors but also considered that signaling of the MAPK–extracellular signal-regulated kinase (ERK) pathway can be activated by various genetic alterations^29^. Indeed, we found that the IL-17 GES also correlates with the presence of other oncogenic mutations in MAPK genes including *NRAS*, *KRAS* and *NF1*. However, we still found several tumor samples with high IL-17 GES, in which we could not detect common MAPK driver mutations by expanded targeted next-generation sequencing genotyping. We assumed that, in such ‘*BRAF*-, *NRAS*- and *NF1*-WT’ samples, the IL-17 pathway might be triggered by alternative regulatory factors such as RORc, STAT3 and NF-κB^30^, possibly resulting from unknown genetic or non-genetic activation. As activation of the MAPK pathway is prevalent in many human cancers^31^, our results may point toward a universal biomarker opportunity for IL-17, not only across different MAPK genotypes, but also across different cancer entities.

We also found patients with melanoma in our tissue and plasma cohorts who did not respond to dual ICI despite a positive *BRAF*-mutant status. Accordingly, the murine YUMM1.7 melanoma model (*BRAF*^V600E^, *Pten*^del^, *Cdkn2a*^del^) also lacks ICI response, which could be explained by the known immune suppressive effects of deleting *Pten* and the associated impaired interferon response and T cell exclusion^32^. Overall, this suggests co-regulation of the IL-17 and MAPK pathways, but the IL-17 pathway is probably not exclusively regulated by oncogenic MAPK activators, nor is the response to dual ICI exclusively related to IL-17 activation. A deeper dissection of the IL-17 regulatory landscape in our tissue discovery cohort is technically not possible because of the limited number of genes that can be detected by the predefined NanoString setup. Future (single-cell) RNA-seq profiling might help to decipher such alternative mechanisms of T_H_17–IL-17 stimulation. The role of IL-17 signaling and T_H_17 cells in cancer progression has been controversially discussed thus far^33^. Studies that evaluated the association between IL-17 and patients’ prognoses are inconsistent across cancer types including melanoma^34–36^. T_H_17 cells and IL-17 are known to have both anti-tumor and pro-tumor effects. However, the underlying mechanism of IL-17 for its anti-tumor or pro-tumor effects in melanoma is not well understood^37^. In mouse models, a few studies supported pro-tumoral activity of IL-17, where knockdown of IL-17 receptor (IL-17R)A or IL-17RC led to decreased formation of B16 melanoma tumors^38,39^. On the other hand, IL-17A-deficient mice have been shown to be susceptible to spontaneous melanoma development^40^ or formation of lung tumors^41^. We found across several published ICI-treated patient cohorts (in total, *n* = 79 dual ICI, *n* = 134 mono anti-PD-1 and *n* = 42 mono anti-CTLA-4 ICI-treated patients^16–19^) that a high baseline IL-17 GES level in melanoma tissue is significantly associated with improved therapy response in dual-ICI-treated but not in mono ICI-treated patients.

High IL-17 signature expression in ICI-treated patient cohorts was additionally positively correlated with higher infiltration of T cells, T_H_17 cells, dendritic cells and neutrophils. This suggests that the role of the pre-existent cytokine milieu and that the associated immune cell populations such as neutrophils, which are commonly considered a negative predictive marker for ICI^42^, might differ depending on the exact therapeutic ICI context. Our in silico results together with the results from in vivo manipulation of IL-17 in two syngeneic melanoma ICI models suggest that the IL-17-associated presence of neutrophils could support the anti-tumor response in patients with melanoma to dual ICI. Likewise, a recent study demonstrated that T cell-mediated tumor elimination follows the recruitment of anti-tumor neutrophils that facilitate the eradication of antigen escape variants in T cell immunotherapies. Furthermore, neutrophil activation was evident in murine but also in human melanoma tumors treated with ICI^43^. Thus, the interplay between T cells and neutrophils might represent an attractive study target to further unravel the immune mechanisms of individual ICI functions on the cellular level in the future.

IL-17A is the hallmark cytokine of T_H_17 cells and is the most potent inducer of downstream cytokines and neutrophil recruitment among IL-17 family members^4^. Therefore, we focused on IL-17A for our cytokine-based approach for outcome stratification of patients with melanoma. In brief, a high baseline IL-17A level in patient plasma samples was indicative of a higher global baseline T_H_17 cytokine profile preceding clinical response to dual ICI in the metastatic setting but not anti-PD-1 monotherapy. It would have also been interesting to analyze IL-17A levels in patient plasma samples from mono anti-CTLA-4-treated patients because of clinical observations made in earlier dose-ranging studies with ipilimumab. However, analysis of a mono anti-CTLA-4-treated patient cohort was not possible due to its current limited use as a monotherapeutic agent in metastatic disease. In the ipilimumab dose-ranging study, *BRAF*-mutant patients had longer median OS than *BRAF*-WT patients with the high (10 mg per kg) but also the standard (3 mg per kg) dose of ipilimumab (33.2 versus 8 months and 19.7 versus 2 months, respectively)^44^. This could indicate that actually ipilimumab is a drug that is predominantly IL-17 responsive also when given as combination in dual ICI. Furthermore, the association between IL-17 and MAPK activation may point to further biomarker opportunities for triple-combination (MAPKi and ICI) therapies, which could be addressed in future studies.

In addition, several studies have shown that the IL-17–T_H_17 pathway predicts the occurrence of irAEs after ICI therapy^45,46^. At the same time, a positive association between irAEs and response to ICI therapy has been found^47,48^. Recent reports now suggest that inhibition of some T_H_17 cytokines, such as IL-6, reduces irAEs without reducing the efficacy of ICI^49^. This differs markedly from the ICI-limiting effects of IL-17 blockade shown in our study and may indicate a more non-linear function within the group of T_H_17 cytokines. In fact, T_H_17 cytokines are pleiotropic and produced by different cell types such as T cells, B cells and macrophages^50^. Therefore, future studies are urgently needed to decipher the multifunctional role of the T_H_17 cytokine network and to understand the immune mechanisms controlling irAE and the response to ICI.

In sum, our data suggest that IL-17A may serve as a biomarker for predicting response to dual-ICI therapy. IL-17A cytokine levels can be measured by common analytical biochemistry assays (for example, enzyme-linked immunosorbent assay (ELISA)) that are easily accessible and applicable in the clinical routine across institutions. To reach the full benefit of cytokine-based therapy selection, several molecular parameters, such as the normal baseline threshold or cytokine concentration dynamics under therapy, need to be investigated in larger prospective cohorts integrating systematic longitudinal sampling protocols.

## Methods

This study complies with all relevant ethical regulations and was approved by the ethics committee of the University Hospital Essen, University of Duisburg-Essen (approval no. 11-4715, 21-9985-BO) and the German animal protection law (Landesamt für Natur, Umwelt und Verbraucherschutz Nordrhein-Westfalen (LANUV NRW) reference no. 81-02.04.2018.A202).

### Analysis of transcriptomic datasets

The discovery cohort consisted of pretreatment tissue samples from 77 treatment-naive *BRAF*^V600E/K^-mutant patients with melanoma from the COMBI-v phase 3 study and 79 treatment-naive *BRAF*-WT patients from the Dermatology Department of the University Hospital Essen^9^. Custom-designed CodeSet (containing 780 genes involved in phenotypic resistance) and the commercially available Immune Panel from NanoString (800 genes involved in immune pathways) were used to generate expression data on the NanoString platform (NanoString Technologies). Clinical parameters of the discovery patient cohort and corresponding gene expression data processing were previously described^9^. The validation cohorts consisted of open-source bulk tumor tissue transcriptomic datasets from the TCGA-SKCM cohort (ICI- and MAPKi-naive patients with melanoma) and ICI- (Liu et al.^17^, phs000452.v3.p1; Van Allen et al.^16^, phs000452.v2.p1; Gide et al.^18^, PRJEB23709; Riaz et al.^19^, GSE91061) or MAPKi- (Long et al.^13^, GSE61992; Rizos et al.^14^, GSE50509; Kakavand et al.^15^, GSE99898) receiving patients with melanoma. Normalized and log_2_ transformed RSEM counts (RNA-seq by expectation maximization) from the TCGA-SKCM cohort were retrieved from the GDAC Firehose (http://gdac.broadinstitute.org). In the SKCM cohort, samples with available mRNA expression and mutation data (*n* = 363) were analyzed. Normalized gene level expression in transcripts per million from the Liu et al.^17^ RNA-seq dataset was downloaded as described in the original study. Raw gene expression counts from the Van Allen et al.^16^ study were normalized using the DESeq2 version 3.17. RNA-seq raw reads from the Gide et al.^18^ and Riaz et al.^19^ studies were downloaded and converted to transcripts per million using the kallisto method. For Kaplan–Meier curves, similar treatment arms from ICI datasets were pooled and analyzed for PFS: Liu et al. dataset, *n* = 47 for anti-CTLA-4 (pretreatment) and anti-PD-1, *n* = 74 for anti-PD-1; Gide et al. dataset, *n* = 32 for anti-CTLA-4 and anti-PD-1, *n* = 41 anti-PD-1 and for OS; Liu et al. dataset, *n* = 47 for anti-CTLA-4 (pretreatment) and anti-PD-1, *n* = 74 for anti-PD-1; Riaz et al. dataset, *n* = 20 for anti-PD-1; Gide et al. dataset, *n* = 32 for anti-CTLA-4 and anti-PD-1, *n* = 40 for anti-PD-1; Van Allen et al. dataset, *n* = 42 for anti-CTLA-4 antibodies. Categorization into low versus high IL-17A–IL-17F GES was carried out separately in each dataset according to the optimal cut point determined in X-tile^28^. Raw gene expression profiling data from the MAPKi datasets featuring a uniform, treatment-naive *BRAF*^V600^-mutant-positive patient cohort by Long et al., Rizos et al. and Kakavand et al. were downloaded from the Gene Expression Omnibus. Count matrices were imported into Partek Flow, where background correction, quantile normalization and log_2_ transformation were carried out. In all validation datasets, the IL-17A–IL-17F GES gene family signature consisted of IL-17 family genes with reliable read counts (expression value > 0 in at least 60% of tumor samples). Gene expression values were summarized into a single GES score without weighing in the normalized dataset. Gene signatures are provided in Supplementary Table 5. Immune cell fraction enrichment analyses from RNA-seq datasets were computed according to the Bindea et al.^51^ immune cell signature using the xCell^52^ algorithm.

### Statistics and reproducibility

The melanoma patient cohort size calculation for cytokine analyses was based on power analysis using the *χ*^2^ statistic, assuming a relative risk of 2.0 between outcome-positive and outcome-negative proportions (type I and II errors at 0.05 and 0.20, respectively). For in vivo experiments, group size was determined based on data from preliminary experiments to detect >20% effect between groups (type I and II errors at 0.05 and 0.20, respectively). In all experiments, a minimum of *n* = 4 mice were used to ensure a balance between statistical needs and animal welfare. For all other experiments, no sample size calculation was performed; however, reproducibility of the method has been demonstrated on a minimum of three biologically independent samples. No patients or cohorts were excluded from the analyses. From public datasets, only the samples with available baseline gene expression, mutational data and clinical annotation were analyzed. Data collection and analysis were performed blinded for human cytokine analyses. Data were not randomized. Normality distribution was assessed by the D’Agostino and Pearson test. Differentially expressed gene set analyses were performed using false discovery rate (FDR) applying a two-stage step-up multiple-test correction with a cutoff of *q* ≤ 0.05 (significant genes are given in Supplementary Table 1). Gene ontology and pathway enrichment analysis was performed on differentially expressed genes using the FDR (*q* ≤ 0.05) approach. Statistical significance was calculated using either the unpaired *t*-test or the Mann–Whitney *U*-test (depending on normality distribution) in two-group comparisons and one-way or two-way ANOVA with multiple-comparison adjustment for more than two groups. Welch’s correction was applied under the unequal standard deviation assumption. Categorical data were analyzed by Fisher’s exact test or the *χ*^2^ test. Kaplan–Meier plots were computed using survival data categorized according to the biomarker threshold determined using X-tile^28^, and curves were compared using the log-rank test. Gene set enrichment analysis was performed using WebGestalt (version 2019)^53^ using KEGG, functional database, with a significance cutoff of FDR ≤ 0.05. All reported *P* values were two tailed, and *P* ≤ 0.05 was considered significant. Effect size was estimated according to Hedge’s *g*. Network prediction and pathway enrichment of differentially expressed proteins were carried out with the STRING database^54^. For statistical and bioinformatic data processing, GraphPad Prism (version 9.5.1), R studio (R-3.6.1 release) and Partek Flow (version 10.0) software was used.

### Cell culture

Human melanoma cell lines with the *BRAF*^V600^ mutation (WM983B, 451Lu, WM9) were maintained at 37 °C in a humidified atmosphere with 5% CO_2_. Cell lines were obtained from the Wistar Institute and cultured in 2% FBS-substituted melanoma medium (‘Tu2%’ medium)^55^. A total of 1 × 10^5^ cells were plated in 6-cm dishes and treated with dabrafenib–trametinib (1 nM, 0.2 nM; Selleckchem) or DMSO (0.1%; AppliChem) for 7 d. Medium containing drugs was replaced after 3 d.

The CM and LN (primary CM and lymph node metastasis: LN, derived from the *ret*-transgenic melanoma model^20^) murine cell lines were cultured in RPMI medium supplemented with 10% FBS. YUMM1.7 (ATCC, CRL-3362) and YUMMER1.7 (Merck, SCC243)^21,56^ cells were cultured in DMEM/F-12 medium supplemented with 10% FBS and 1% NEAA. A total of 1 × 10^5^ cells were plated in 6-cm dishes and treated with 25 ng ml^−1^ rm-IL-17A or solvent (water) for 48 h. Conditioned medium was collected and centrifuged, and supernatants were used for short-term culturing of naive BM neutrophils and for cytokine assays.

### Real-time quantitative PCR

Total RNA was isolated from cell pellets using the RNeasy Mini Kit according to the manufacturer’s protocol (Qiagen). qPCR was carried out on the StepOnePlus (Thermo Fisher Scientific) system. Each reaction was set up in technical replicates with wells containing 10 ng total RNA, 10 µM primer pairs, 1× Luna Universal One-Step Reaction Mix and 1× Luna WarmStart RT Enzyme Mix (Luna Universal One-Step RT–qPCR Kit, New England Biolabs). Results were analyzed with StepOne software version 2.3 (Thermo Fisher Scientific). mRNA expression was calculated using the 2^−ΔΔCt^ method^57^ and normalized to the geometric mean of housekeeping genes *RNA18S*, *POLR2A* or *GAPDH*. Each experiment was repeated at least twice. Primer sequences are listed in Supplementary Table 2.

### In vivo studies

For all in vivo studies, 8–10-week-old female C57BL/6N or C57BL/6J mice were used. To study tumor growth kinetics under ICI and combination treatments, 5 × 10^5^ CM cells (derived from the spontaneous MT/*ret* mouse model, *BRAF*-WT, ICI sensitive)^20,58^, 1.5 × 10^6^ YUMMER1.7 (*BRAF*-mutant, ICI-sensitive)^21^ or 1 × 10^5^ YUMM1.7 (*BRAF*-mutant, ICI-resistant)^56^ mouse melanoma cells were injected subcutaneously in PBS (YUMM1.7, YUMMER1.7) or in a 1:1 mixture of PBS with Matrigel (CM). The following treatments in different combinations (total injection volume of 200 µl) were administered by intraperitoneal injection: control IgG (IgG2a isotype control clone 2A3, BioXCell, 10 mg per kg body weight, 3× per week) anti-CTLA-4 antibody (anti­-mouse CTLA-4 clone 9D9, BioXCell, 8 mg per kg body weight, 3× per week), anti-PD-1 antibody (anti­-mouse PD­1 clone RMP1-14, BioXCell, 10 mg per kg body weight, 3× per week), rm-IL-17A (IL-17A mouse recombinant, Prospec, 0.01 mg per kg body weight, daily), α-IL-17A (Ultra-LEAF purified anti-mouse IL-17A antibody clone TC11-18H10.1, BioLegend, 4 mg per kg body weight, 3× per week), anti-Ly6G antibody (anti-mouse Ly6G clone 1A8, Leinco Technologies, 4 mg per kg body weight, 3× per week, starting from day −2) according to the treatment schedule summarized in the schematics above the corresponding growth curves. Pretreatment with ICI was carried out for the CM model^58^. Mice were randomized to different combinatorial treatment groups when tumors became palpable. Treatment continued until tumors had reached the maximal volume (not exceeding 1,500 mm^3^) or became ulcerated. Tumor growth kinetics were analyzed in long-term experiments, while short-term experiments (end of treatment on day 9 or day 12) were set up to analyze immune infiltration by multiplex immunofluorescence or flow cytometry and serum cytokine profiles by multiplex cytokine array. Tumor volume was assessed by caliper measurement (calculated as *W* × *W* × *L* ÷ 2). At the end of the treatment, animals were killed, and tumor and blood samples were collected. Tumor samples were fixed in formalin for histological assessment and immunostaining. Blood samples were collected by cardiac puncture in Microvette 100 Serum tubes (Sarstedt). Serum was separated by a standard centrifugation protocol and stored at –80 °C until analysis. Serum samples with substantial hemolysis from red blood cells were excluded from cytokine analyses. TANs were isolated by flow cytometry (CD45^+^CD11B^+^Ly6G^+^ sorted fraction) from single-cell suspensions derived from tumors 8 d (mean tumor volume, ~250 mm^3^) after subcutaneous injection with CM or LN cells (5 × 10^5^) in 8–10-week-old C57BL/6N mice. For proteomic analysis, proteins were liberated by cell lysis. After sample purification and tryptic digestion, peptides were analyzed by LC-MS/MS. All animal experiments were performed in accordance with institutional and national guidelines and regulations. Ethical approval was provided by the local state authority LANUV NRW in compliance with the German animal protection law (reference number 81-02.04.2018.A202).

### Immune cell isolation and in vitro analysis

Naive BM neutrophils were isolated from femurs of 10-week-old female C57BL/6N mice with the mouse Neutrophil Isolation Kit (Miltenyi) using anti-biotin microbead technology according to the instruction manual by the manufacturer. Purity was confirmed by flow cytometry and the >90% CD45^+^CD11b^+^Ly6G^+^ fraction was accepted for downstream analysis. Isolated neutrophils were cultured short-term (24 h) in either RPMI with 10% FBS or conditioned medium derived from untreated or rm-IL-17A (25 ng ml^−1^ mouse recombinant IL-17 Prospec)-treated CM mouse melanoma cells. In some experiments, α-IL-17A (5 µg ml^−1^ Ultra-LEAF purified anti-mouse IL-17A antibody clone TC11-18H10.1, BioLegend) was added to the culture medium. Cell culture supernatants were centrifuged and used for cytokine analysis. Cell pellets were used for RNA isolation and downstream qPCR analysis. Primer sequences are provided in Supplementary Table 2.

CD8^+^ T cells were isolated from spleen tissues of 10-week-old female C57BL/6N mice using the mouse CD8a^+^ T Cell Isolation Kit (Miltenyi) according to the instruction manual by the manufacturer. Purity was confirmed by flow cytometry, and the >90% CD45^+^CD3^+^CD8^+^ fraction was accepted for downstream analysis. A total of 1.5 × 10^5^ CD8^+^ T cells were plated in migration medium (RPMI with 1% BSA) in the upper chamber of a Boyden chamber (6.5-mm Transwell with 5.0 µm Pore Polycarbonate Membrane Insert, Corning), and 600 µl conditioned media from different treatments were added to the bottom chamber. The different conditioned media were from untreated or rm-IL-17A (25 ng ml^−1^ mouse recombinant IL-17 Prospec)-treated CM melanoma cells with or without the downstream culturing step with BM neutrophils. In some experiments, α-IL-17A (5 µg ml^−1^ Ultra-LEAF purified anti-mouse IL-17A antibody clone TC11-18H10.1, BioLegend) was added to the upper chamber for the duration of the migration. Serum-free medium was used as the negative control, and 200 ng ml^−1^ mouse recombinant CXCL10 diluted in PBS with 1% BSA was used as the positive control. After 12–18 h of migration at 37 °C in a humidified atmosphere with 5% CO_2_, living (Trypan blue-negative) migrated cells were counted under the microscope using a Neubauer chamber.

### Multiplex immunofluorescence

Multiplex immunofluorescence staining of 4-µm, formalin-fixed paraffin-embedded mouse tumor tissue sections (three mice for each combination drug treatment group) was executed. Deparaffinization and antigen retrieval was performed using the Dako PT Link heat-induced antigen retrieval solution with high-pH (pH 9) target retrieval solution (Dako). Next, each tissue slide was stained in three consecutive rounds of antibody staining, using the Opal Multiplex IHC Kit (Akoya). The slides were washed with Tris-buffered saline containing 0.05% Tween-20, and the microwave treatment was performed in Tris–EDTA buffer (pH 9). If the antibody host species were neither rabbit nor mouse (as provided in the kit), a horseradish peroxidase-conjugated secondary antibody for mouse or hamster (Jackson ImmunoResearch) was used at 1:1,000 in antibody diluent (Akoya Biosciences), followed by TSA visualization with Opal fluorophores (Akoya Biosciences) diluted in 1× Plus Amplification Diluent (Akoya Biosciences). The immunofluorescence panels consisted of melan A (EPR20380, 1:1,000, Abcam), Ly6G (RB6-8C5, 1:100, BioLegend), CD8a (C8/144B, 1:100, BioLegend), CD11c (N418, 1:100, BioLegend), CD4 (RM4-5, 1:100, BioLegend) and IL-17A (TC11-18H10.1, 1:100, BioLegend) primary antibodies. Nuclei were stained with DAPI. Imaging was performed with Zeiss Axio Scan (×20 objective) microscopy. The relative contribution of immune cells was calculated by quantitating the background-corrected mean fluorescence intensity of each marker at five random fields per tumor tissue and normalized to DAPI values. Quantitation was performed with ImageJ Fiji software following guidelines by Shihan et al.^59^.

### Flow cytometry analysis

Tissues were digested using the Mouse Tumor Dissociation Kit (Miltenyi) on the gentleMACS device (Miltenyi) according to the manufacturer’s instructions. Red blood cell lysis buffer (BioLegend) was used to remove red blood cells. After washing with PBS, cells were incubated with TruStain fcX anti-mouse CD16/32 receptor blocking agent (BioLegend) diluted in Cell Staining Buffer (BioLegend) for 20 min at 4 °C. After washing, Zombie NIR cell viability dye (1:2,000, BioLegend) was added and incubated for 20 min at 4 °C. To assess immune cell composition, the following antibodies were added for 30 min at 4 °C: for lymphocytes, anti-CD45 PerCP Cy5.5 (30-F11, 1:100), anti-CD3 FITC (17A2, 1:100), anti-CD4 PB (RM4-5, 1:100), anti-CD8a BV 510 (53-6.7, 1:100) and anti-granzyme B AF 647 (GB11, 1:100); for macrophages, anti-CD45 PerCP Cy5.5 (30-F11, 1:100), anti-CD11B PB (M1/70, 1:100), anti-CD11C AF 488 (N418, 1:100), anti-Ly6C AF 647 (HK1.4, 1:100) and anti-Ly6G PE (1A8, 1:100), all from BioLegend. Granzyme B was added after surface staining was completed and after fixation–permeabilization (Fixation Buffer, BioLegend; 10× Intracellular Staining Perm Wash Buffer, BioLegend). Subsequently, samples were washed twice before data acquisition on the BD Aria III flow cytometer. The gating strategy is shown in Extended Data Fig. 3b.

### Human patient-derived tumor fragments

PDTF cultures were performed as previously described^22^. In short, tumor specimens were collected from three patients with melanoma undergoing surgery. The tissue was manually dissected into fragments of 1–2 mm^3^ and cryopreserved in freezing medium (FCS supplemented with 10% DMSO) until use. Tumor fragments were thawed and embedded in artificial matrix (Cultrex UltiMatrix (Bio-Techne, 2 mg ml), rat tail collagen I (Corning, 1 mg ml^−1^), sodium bicarbonate (Sigma-Aldrich, 1.1%) and DMEM tumor medium (Thermo Fisher Scientific) supplemented with 1 mM sodium pyruvate (Sigma-Aldrich), 1× MEM nonessential amino acids (Sigma-Aldrich), 2 mM l-glutamine (Thermo Fisher Scientific), 10% FBS and 1% penicillin-streptomycin) in a 96-well plate, using 8–10 fragments for each treatment condition. For PDTF stimulation, the medium was supplemented with anti-PD-1 (10 µg ml^−1^, nivolumab, Bristol Myers Squibb), anti-CTLA-4 (10 µg ml^−1^, ipilimumab, Bristol Myers Squibb) and α-IL-17A (10 µg ml^−1^, clone BL168, BioLegend) antibodies. After 48 h of incubation at 37 °C, supernatants were collected, and chemokine and cytokine secretion was assessed using the LEGENDplex Human Th Cytokine and Human Proinflammatory Chemokine assays, according to the manufacturer’s protocol.

### Patient samples

Plasma samples (*n* = 117) from 70 patients with melanoma who received first-line ipilimumab plus nivolumab and plasma samples (*n* = 76) from 51 patients with melanoma who received first-line nivolumab or pembrolizumab were collected at therapy baseline and before the first staging evaluation (median, week 9; range, 2–12 weeks). All patients were treated at the Department of Dermatology of the University Hospital Essen in standard-of-care or clinical trial settings. Serum samples (*n* = 89) from patients with melanoma who received ipilimumab plus nivolumab (*n* = 45) or nivolumab or pembrolizumab (*n* = 44) were collected at therapy baseline across four independent centers (Tübingen, Mannheim and Essen in Germany; St. Gallen in Switzerland). Baseline clinicopathological characteristics are given in Supplementary Tables 3 and 4. Radiologic tumor response was evaluated by an independent radiologist according to RECIST criteria. Patients with complete response and partial response were classified as responders, while those with mixed response and progressive disease were classified as non-responders. For the Essen cohorts, human biological samples and related data were provided by the Westdeutsche Biobank Essen (WBE/SCABIO, University Hospital Essen, University of Duisburg-Essen, Essen, Germany; approval nos. 11-4715, 21-9985-BO). The samples were prospectively collected and archived at the local WBE/SCABIO biobank according to institutional informed consent procedures and retrospectively evaluated for this study. Serum samples in the validation cohorts were collected in compliance with the ethical regulations of the respective institutions, and approval was provided by the ethical committee of Tübingen University Medical Center (490/2014 B01, 089/2021A), the Ethical Committee II of Heidelberg University (2010-318N-MA) and Ethikkommission Ostschweiz (EKOS 16/079). Resected tumor samples were collected from patients with melanoma undergoing surgical treatment at the Netherlands Cancer Institute (NKI-AVL), the Netherlands. The study was approved by the institutional review board of the NKI-AVL (CFMPB484) and executed in compliance with ethical regulations. All patients consented to the research usage of material not required for diagnostics via prior informed consent.

### Secreted cytokine profiling

Secreted levels of human or mouse IL-17A in plasma or serum were determined according to the manufacturer’s instructions (LEGEND MAX Human IL-17A ELISA Kit, LEGEND MAX Mouse IL-17A ELISA Kit, BioLegend). For human samples, plasma samples from patients with psoriasis were used as internal reference controls. For multiplex quantification of cytokines, the bead-based LEGENDplex panels (Human Th17 7-plex Panel; Human Th 12-plex Panel, Mouse Th17 7-plex Panel; IL-1β, IL-23 and IL-12p70 from the Inflammation Panel 1; granzyme A and granzyme B from the CD8/NK Panel, predefined and custom-designed mix-and-match system from BioLegend) were used according to the manufacturer’s instructions. Flow cytometry reading was performed on the FACSAria III (BD). Mean fluorescence intensity values were recorded using LEGENDplex analysis software (version 2021.07.01), and cytokine concentrations (pg ml^−1^) were interpolated from a five-parameter logistic non-linear curve model using a separate standard curve for each cytokine. For prognostic stratification of IL-17A plasma levels, an optimal cut point was determined in each dataset separately using X-tile^28^.

### Reporting summary

Further information on research design is available in the Nature Portfolio Reporting Summary linked to this article.

### Supplementary information

Reporting Summary

Supplementary Tables Supplementary Tables 1–5.

### Source data

Source Data Fig. 1 Statistical source data.

Source Data Fig. 2 Statistical source data.

Source Data Fig. 2 Imaging source data.

Source Data Fig. 3 Statistical source data.

Source Data Fig. 4 Statistical source data.

Source Data Fig. 5 Statistical source data.

Source Data Fig. 6 Statistical source data.

Source Data Extended Data Fig. 1 Statistical source data.

Source Data Extended Data Fig. 2 Statistical source data.

Source Data Extended Data Fig. 2 Imaging source data.

Source Data Extended Data Fig. 3 Statistical source data.

## Data Availability

Previously published RNA-seq data that were reanalyzed here are available under accession codes phs000452.v3.p1 (Liu et al.^17^), phs000452.v2.p1 (Van Allen et al.^16^), PRJEB23709 (Gide et al.^18^), GSE91061 (Riaz et al.^19^), GSE61992 (Long et al.^13^), GSE50509 (Rizos et al.^14^) and GSE99898 (Kakavand et al.^15^). Data from the discovery cohort (Brase et al.^9^) that were derived from the COMBI-v trial (Novartis) were obtained directly from the authors with the permission of Novartis. Novartis is committed to sharing with qualified external researchers access to patient-level data and supporting clinical documents from eligible studies. Requests are reviewed and approved by an independent review panel on the basis of scientific merit. All data provided are anonymized to respect the privacy of patients who have participated in the trial in line with applicable laws and regulations. This trial data availability is according to the criteria and process described at https://clinicalstudydatarequest.com. Human melanoma RNA-seq data were derived from the TCGA Research Network: http://cancergenome.nih.gov/. All other data supporting the findings of this study are available from the corresponding author on reasonable request. Source data are provided with this paper.

## Code availability

Only open-source software was used for this study, and no custom codes were generated for RNA-seq analysis.
