# Author Correction: Interleukin 17 signaling supports clinical benefit of dual CTLA-4 and PD-1 checkpoint inhibition in melanoma

**DOI:** 10.1038/s43018-023-00632-w

**Published:** 2023-08-14

**Authors:** Renáta Váraljai, Lisa Zimmer, Yahya Al-Matary, Paulien Kaptein, Lea J. Albrecht, Batool Shannan, Jan C. Brase, Daniel Gusenleitner, Teresa Amaral, Nina Wyss, Jochen Utikal, Lukas Flatz, Florian Rambow, Hans Christian Reinhardt, Jenny Dick, Daniel R. Engel, Susanne Horn, Selma Ugurel, Wiebke Sondermann, Elisabeth Livingstone, Antje Sucker, Annette Paschen, Fang Zhao, Jan M. Placke, Jasmin M. Klose, Wolfgang P. Fendler, Daniela S. Thommen, Iris Helfrich, Dirk Schadendorf, Alexander Roesch

**Affiliations:** 1 Department of Dermatology, University Hospital Essen, West German Cancer Center, University Duisburg-Essen and the German Cancer Consortium (DKTK), Essen, Germany; 2 Division of Molecular Oncology and Immunology, the Netherlands Cancer Institute, Amsterdam, the Netherlands; 3 Novartis Pharma AG, Basel, Switzerland; 4 Novartis Institutes for BioMedical Research, Inc., Cambridge, MA USA; 5 Department of Dermatology, University Hospital of Tübingen, Tübingen, Germany; 6 Institute of Immunobiology, Kantonsspital St. Gallen, Switzerland, Switzerland; 7 Skin Cancer Unit, German Cancer Research Center (DKFZ), Heidelberg, Germany; 8 Department of Dermatology, Venereology and Allergology, University Medical Center Mannheim, Ruprecht Karls University of Heidelberg, Mannheim, Germany; 9 DKFZ Hector Cancer Institute at the University Medical Center Mannheim, Mannheim, Germany; 10 Department of Applied Computational Cancer Research, Institute for AI in Medicine (IKIM), University Hospital Essen, Essen, Germany; 11 Department of Hematology and Stem Cell Transplantation, University Hospital Essen, Essen, Germany; 12 Center for Medical Biotechnology (ZMB), University of Duisburg-Essen, Essen, Germany; 13 Department of Immunodynamics, Institute of Experimental Immunology and Imaging, University Hospital Essen, Essen, Germany; 14 Rudolf Schönheimer Institute of Biochemistry, Medical Faculty, University of Leipzig, Leipzig, Germany; 15 Department of Nuclear Medicine, University Hospital Essen, University of Duisburg-Essen, Essen, Germany; 16 Department of Dermatology and Allergology, Ludwig Maximilian University Munich, Munich, Germany; 17 NCT West, Campus Essen and University Alliance Ruhr, Research Center One Health, University Duisburg-Essen, Essen, Germany

**Keywords:** Melanoma, Cancer immunotherapy, Tumour immunology, Cancer

Correction to: *Nature Cancer* 10.1038/s43018-023-00610-2. Published online 31 July 2023.

In the version of this article initially published, the description of the proteomic analysis was incorrectly termed reverse phase protein array (RPPA) instead of liquid chromatography–mass spectrometry/mass spectrometry (LC-MS/MS). The Figure 3d schematic and Figure 3d,e legends as well as the Extended Data Figure 3 legend have been updated, and the Results section “The IL-17-associated cellular microenvironment in dual ICI” and Methods section “In vivo studies” have been corrected accordingly. The error does not affect the results or conclusions of this article, and we apologize for any confusion this may have caused.

