# Supplementary material for: Interleukin 17 signaling supports clinical benefit of dual CTLA-4 and PD-1 checkpoint inhibition in melanoma
Source: Nat Cancer. 2023 Jul 31;4(9):1292–308. doi: 10.1038/s43018-023-00610-2 (PMC10518254; doi:10.1038/s43018-023-00610-2)
Supplement: Supplementary file 1 — Reporting Summary [file 43018_2023_610_MOESM1_ESM.pdf]

nature portfolio

Corresponding author(s): Alexander Roesch
Last updated by author(s): Jun 13, 2023

Reporting Summary

Nature Portfolio wishes to improve the reproducibility of the work that we publish. This form provides structure for consistency and transparency in reporting. For further information on Nature Portfolio policies, see our Editorial Policies and the Editorial Policy Checklist.

Statistics

For all statistical analyses, confirm that the following items are present in the figure legend, table legend, main text, or Methods section.

n/a	Confirmed	
☐	☒	The exact sample size (*n*) for each experimental group/condition, given as a discrete number and unit of measurement
☐	☒	A statement on whether measurements were taken from distinct samples or whether the same sample was measured repeatedly
☐	☒	The statistical test(s) used AND whether they are one- or two-sided<br>*Only common tests should be described solely by name; describe more complex techniques in the Methods section.*
☒	☐	A description of all covariates tested
☐	☒	A description of any assumptions or corrections, such as tests of normality and adjustment for multiple comparisons
☐	☒	A full description of the statistical parameters including central tendency (e.g. means) or other basic estimates (e.g. regression coefficient) AND variation (e.g. standard deviation) or associated estimates of uncertainty (e.g. confidence intervals)
☐	☒	For null hypothesis testing, the test statistic (e.g. *F*, *t*, *r*) with confidence intervals, effect sizes, degrees of freedom and *P* value noted<br>*Give P values as exact values whenever suitable.*
☒	☐	For Bayesian analysis, information on the choice of priors and Markov chain Monte Carlo settings
☒	☐	For hierarchical and complex designs, identification of the appropriate level for tests and full reporting of outcomes
☐	☒	Estimates of effect sizes (e.g. Cohen's *d*, Pearson's *r*), indicating how they were calculated

Our web collection on statistics for biologists contains articles on many of the points above.

Software and code

Policy information about availability of computer code

Data collection	The TCGA SKCM gene expression data was retrieved from the GDAC Firehose (http://gdac.broadinstitute.org) site.
Data analysis	The following softwares were used: GraphPad Prism v.9.5.1 for statistical testing and visualization, Partek Flow v.10.0 for microarray data processing and count normalization, ImageJ v.1.50i for image quantification, R v.3.6.1 for RNA-Seq data kallisto normalization, DeSeq2 v.3.17 for normalization, StepOnePlus v.2.3 for qRT-PCR data analyses, ZEN v.3.1 (blue edition) for fluorescent image processing, FlowJo v.10.8.1 for flow cytometry data, X-tile v.3.6.1 for optimal cutpoint determination for survival curves, WebGestalt v.2019 (http://www.webgestalt.org/) for pathway and GSEA analysis, LegendPlex analysis software v. 2021. 07.01 (https://legendplex.qognit.com/) for MFI determination of cytokine arrays, STRING v.11.5 database (https://string-db.org/) for protein-protein interaction, xCell v.2020 (https://xcell.ucsf.edu/) for immune deconvolution. Hedges' g was calculated after the formula by Hedges and Olkin (Hedges, L. V. & Olkin, I. 1985: Statistical methods for meta-analysis. Orlando, FL: Academic Press).

For manuscripts utilizing custom algorithms or software that are central to the research but not yet described in published literature, software must be made available to editors and reviewers. We strongly encourage code deposition in a community repository (e.g. GitHub). See the Nature Portfolio guidelines for submitting code & software for further information.

Data

Policy information about availability of data

All manuscripts must include a data availability statement. This statement should provide the following information, where applicable:

- Accession codes, unique identifiers, or web links for publicly available datasets
- A description of any restrictions on data availability
- For clinical datasets or third party data, please ensure that the statement adheres to our policy

Previously published RNA-Seq data that were re-analysed here are available under accession code phs000452.v3.p1 [Liu et al 17]; phs000452.v2.p1 [Van Allen et al ref. 16]; PRJEB23709 [Gide et al ref. 18]; GSE91061 [Riaz et al ref. 19]; GSE61992 [Long et al ref. 13]; GSE50509 [Rizos et al ref. 14]; GSE99898 [Kakavand et al ref. 15]. Data from the discovery cohort [Brase et al ref. 9] was obtained directly from the authors with permission of Novartis. The human melanoma RNA-Seq data were derived from the TCGA Research Network: http://cancergenome.nih.gov/. Source data for Fig. 1a, 3e, and Extended Data Fig. 1a (KEGG database, release no. 99) and 1b have been provided as Source Data files. All other data supporting the findings of this study are available from the corresponding author on reasonable request.

Human research participants

Policy information about studies involving human research participants and Sex and Gender in Research.

Reporting on sex and gender	All human research participants were included in the study regardless of sex and gender. In the total plasma/serum cohort (Figure 4, S4) the sex distribution was 58% males and 42% females.
Population characteristics	Plasma cohort: n = 70 metastatic melanoma patients who received first-line ipilimumab plus nivolumab (median age 60 yrs; n = 45 male, n = 25 female; n = 27 BRAF V600 positive, n = 43 BRAF V600 negative) and n = 51 metastatic melanoma patients who received first-line nivolumab or pembrolizumab (median age 71 yrs; n = 28 male, n = 23 female; n = 11 BRAF V600 positive, n = 38 BRAF V600 negative, n = 2 unknown) were included in this study.<br>Serum cohort: n = 45 metastatic melanoma patients who received ipilimumab plus nivolumab (median age 62 yrs; n = 29 male, n = 16 female; n = 17 BRAF V600 positive, n = 25 BRAF V600 negative, n = 3 unknown) and n = 44 metastatic melanoma patients who received nivolumab or pembrolizumab (median age 71 yrs; n = 20 male, n = 24 female; n = 12 BRAF V600 positive, n = 31 BRAF V600 negative, n = 1 unknown) were included in this study.<br>Patient-derived tumor fragments (PDTFs): Clinical data of this cohort are described by the original authors [references 23,24]. This study also included analysis of previously published melanoma cohorts [as described in section Software and code/Data collection]. Clinical data of these cohorts are described by the authours in their respective publications [references 14-20].
Recruitment	The samples analyzed in this study were obtained through biobanking procedures as outlined below. Patients were not recruited specifically for this study. Patients did not receive compensation.<br>All patients who received immunotherapy and consented to institutional biobanking procedures were identified by treating physicians at the respective institutions.<br>Patients in the plasma cohort were treated at the Department of Dermatology of the University Hospital Essen in Germany in standard-of-care or clinical trial settings.<br>Patients in the serum cohort were treated at dermatology departments at the University Hospital of Tübingen (Germany), University Medical Center Mannheim (Germany), Kantonsspital St. Gallen (Switzerland), and University Hospital Essen (Germany) in standard-of-care or clinical trial settings.<br>PDTFs: Human resected tumor samples were collected from patients with melanoma undergoing surgical treatment at the Netherlands Cancer Institute (NKI-AVL).
Ethics oversight	Plasma and serum Essen cohorts: human biological samples and related data were provided by the Westdeutsche Biobank Essen (WBE/SCABIO) and approval was provided by the University Hospital Essen, University of Duisburg-Essen, Essen, Germany (approval no. 11-4715, 21-9985-BO) according to institutional informed consent procedures.<br>Additional serum cohorts: sample were collected in compliance with the ethical regulations of the respective institutions' and approval was provided by the Ethical committee of Tübingen University Medical Center (490/2014 B01, 089/2021A), the Ethical committee II of Heidelberg University (2010-318N-MA) and Ethikkommission Ostschweiz (EKOS 16/079).<br>PDTFs: resected tumor samples were collected from patients with melanoma undergoing surgical treatment at the Netherlands Cancer Institute (NKI-AVL), The Netherlands. The study was approved by the institutional review board of the NKI-AVL (CFMPB484) and executed in compliance with the ethical regulations. All patients consented to the research usage of material not required for diagnostics via prior informed consent.

Note that full information on the approval of the study protocol must also be provided in the manuscript.

Field-specific reporting

Please select the one below that is the best fit for your research. If you are not sure, read the appropriate sections before making your selection.

☒ Life sciences ☐ Behavioural & social sciences ☐ Ecological, evolutionary & environmental sciences

For a reference copy of the document with all sections, see nature.com/documents/nr-reporting-summary-flat.pdf

Life sciences study design

All studies must disclose on these points even when the disclosure is negative.

Sample size	Human plasma and serum samples: the melanoma patient cohort size calculation for cytokine assays was based on power analysis using Chi-squared statistic assuming a relative risk of 2.0 between outcome positive and outcome negative proportions (type I/II errors at 0.05 and 0.20, respectively).<br>Mice: group size was determined based on data from preliminary experiments to detect >20% effect between groups (type I/II errors at 0.05 and 0.20, respectively). In all experiments a minimum of n = 4 mice were used to ensure a balance between statistical needs and animal welfare.<br>Sample size was calculated on https://sample-size.net/.<br>For all other experiments, including in vitro experiments, no sample size calculation was performed, however, reproducibility of the method has been demonstrated on minimum three biologically independent samples. In addition, successful replication experiments were considered enough to demonstrate sufficient sample size to identify consistent differences between groups.
Data exclusions	Public gene expression profiling datasets: only the samples with available baseline gene expression, mutational data and clinical annotation were analyzed.<br>In the TCGA SKCM cohort, only one sample/patient was analyzed for survival analysis (in case of patients who donated multiple samples).<br>In the MAPKi datasets (GSE61992, GSE50509, GSE99898) where replicate tumors of the same tumor lesions were profiled, we analyzed the sample with higher expression value (in general there were minor expression differences for replicate tumors). Exact patient numbers are given in Materials and Methods and reflected in figures (e.g. in Kaplan Meier plots) or in figure legends (Figure 1f-l).<br>Murine cytokine measurements: serum samples with significant hemolysis from red blood cells and samples which yielded less than 50 microliter serum (amount needed for ELISA assay) were not analyzed for cytokine levels (Figure 2b, Extended Figure 2d).<br>Flow cytometry: each murine tumor sample was divided equally to run two staining protocols (lymphocyte and myeloid panels, see below under Flow Cytometry section) simultaneously, where n=1 tumor (out of n = 5) yielded in less than 20,000 viable cells, thus could not be included in the lymphocyte panel (Figure 4c).
Replication	Individual dots represent individual patients/mice/cell lines in figures and these are described in the figure legends.<br>In all in vitro experiments technical replicates were set up (n = 2 to 10; see list below) and were reproduced independently at least twice. All attempts and replication were successful.<br><br>RT-qPCR: minimum n = 3 biological replicates, minimum n = 2 to 3 technical replicates were set up.<br><br>IF staining and quantification: n = 2 mouse tumors (biological replicates) was used for IHC. Shown in Figure 2g and Extended Figure 2b is 1 representative tumor/treatment group. Consecutive tissue slices were stained for the three different antibody panels. For quantification n = 10 random fields/whole tumor area was digitally quantified.<br><br>Cytokine assays (ELISA and LegendPlex): technical replicates (n = 2) were set up following manufacturer's instruction. Samples derived from individual patients (total n = 210), or mice (minimum n = 4 mice/group/experiment) were regarded as biological replicates.<br><br>In vivo experiments:<br>In general, treatment effects were tested using two independent mouse models. Where possible, different mouse strains were used. The results were reproducible between mouse models and strains, these are detailed below:<br>Figure 2: using the CM mouse model in C57BL/6N mice, the same treatment groups and doses were set up in two independent experiments (Figure 2a and Figure 2g for long or short-term growth kinetics). Figure 2a was replicated using an independent mouse melanoma model (YUMM1.7) in C57BL/6J mice (Extended Figure 2c).<br>Figure 4: the same treatment groups and doses were set up in two independent experiments using two independent mouse models (CM and YUMMER1.7) in C57BL/6N mice (Figure 4a and 4b).
Randomization	In vitro experiments: Mouse tumors were sectioned and subjected to IF staining in a consistant manner without specific allocation. When quantifying the images for specific marker expression, microscopy fileds to be quantified were randomly chosen adhering to the same area size within the whole tumor area. Background correction was done separately for each marker and results were normalized to their respective DAPI controls.<br>For other in vitro experiments, no relevant differing covariates could be identified, thus there was no need for randomization. All samples were processed/measured in a consistent manner.<br>In vivo experiments: mice were randomly grouped in different cages. Within each cage, mice were randomized for different ICI therapies. When tumors became palpable (typically on day 5), ICI receiving mice were further randomized to different combination therapies.<br>Patient samples were also not randomized since the clinical variable (e.g. genotype or reponse to therapy) analyzed determined the group. Data was not randomized.
Blinding	In vitro experiments: human cytokine analyses (ELISA and LegendPlex multiplex cytokine array) on plasma and serum samples were done in a blinded fashion. Investigators who performed cytokine measurement were blinded to clinical response data which was provided by independent medical researchers after completion of sample measurement.<br><br>In vivo experiments: complete blinding was not possible, even though therapy administration was done following eartags. However, during tumor size measurement the investigator who measured had no knowledge of the treatment group and cage number which was picked by a second assisting investigator.<br><br>For all other experiments (in vitro, bioinformatics) blinding was not possible since the person who performed the experiment had to have knowledge of the condition applied (i.e. treatment with drug vs control) or group to be analyed. However, when possible, a second researcher confirmed the results.

Reporting for specific materials, systems and methods

We require information from authors about some types of materials, experimental systems and methods used in many studies. Here, indicate whether each material, system or method listed is relevant to your study. If you are not sure if a list item applies to your research, read the appropriate section before selecting a response.

Materials & experimental systems

n/a	Involved in the study
☐	☒ Antibodies
☐	☒ Eukaryotic cell lines
☒	☐ Palaeontology and archaeology
☐	☒ Animals and other organisms
☒	☐ Clinical data
☒	☐ Dual use research of concern

Methods

n/a	Involved in the study
☒	☐ ChIP-seq
☐	☒ Flow cytometry
☒	☐ MRI-based neuroimaging

Antibodies

Antibodies used

IF:
Melan A clone EPR20380, Abcam #ab210546, 1:1000
Ly6G clone RB6-8C5, BioLegend #108401, 1:100
CD8a clone C8/144B, BioLegend #372902, 1:100
CD11c clone N418, BioLegend #117301, 1:100
CD4 clone RM4-5, Biolegend # 100505, 1:100
IL-17A clone TC11-18H10.1, BioLegend # 506901, 1:100
Peroxidase AffiniPure Mouse Anti-Rat IgG (H+L), Dianova, #212-035-082, 1:1000
Peroxidase AffiniPure Goat Anti-Armenian Hamster IgG (H+L), Dianova, #127-035-160, 1:1000

In vivo:
anti-mouse CTLA-4 clone 9D9, BioXCell #BE0164, 8 mg/kg body weight
anti-mouse PD-1 clone RMP1-14, BioXCell #BE0146, 10 mg/kg body weight
IgG2a isotype control clone 2A3, BioXCell #BE0089, 10 mg/kg body weight
Ultra-LEAF™ purified anti-mouse IL-17A antibody clone TC11-18H10.1, BioLegend #506946, 4 mg/kg body weight
anti-mouse Ly6G clone 1A8, Leinco Technologies #L280, 4 mg/kg body weight
IL-17A mouse recombinant, Prospec #CCYT-378, 0.01 mg/kg body weight

Ex vivo:
anti-human IL-17A clone BL168, Biolegend #512302, 10 µg/mL final concentration
anti- PD-1: Nivolumab, Bristol-Myers Squibb #BMS-936558, 10 µg/mL final concentration
anti CTLA-4: Ipilimumab, Bristol-Myers Squibb #BMS-986288, 10 µg/mL final concentration

Flow cytometry:
Lymphocyte panel:
anti-CD45 PerCP Cy5.5, clone 30-F11, BioLegend #103131, 1:100
anti-CD3 FITC, clone 17A2, BioLegend 3100203, 1:100
anti-CD4 Pacific Blue, clone RM4-5, BioLegend #100534, 1:100
anti-CD8a Bright Violet 510, clone 53-6.7, BioLegend #100752, 1:100
anti-Granzyme B Alexa Fluor 647, BioLegend #515405, clone GB11, 1:100
Myeloid panel:
anti-CD45 PerCP Cy5.5, clone 30-F11, BioLegend #103131, 1:100
anti-CD11b Pacific Blue, clone M1/70, BioLegend #101223, 1:100
anti-CD11c Alexa Fluor 488, clone N418, BioLegend #117313, 1:100
anti-Ly6C Alexa Fluor 647, clone HK1.4, BioLegend #128009, 1:100
and anti-Ly6G PE, clone 1A8, BioLegend #127607, 1:100

Validation

All antibodies were purchased from established companies with well-documented specificites and experimental applications (paper references, data sheets). All antibodies were validated by the supplier.

Website, Host species, Reactivity, Suitability
IF:
https://www.biolegend.com/en-us/products/purified-anti-mouse-ly-6g-ly-6c-gr-1-antibody-462 Rat Mouse IF, Flow Cyt.,WB,IHC, IP
https://www.biolegend.com/en-us/products/purified-anti-human-cd8a-antibody-13983 Mouse Human, Mouse, Rat IHC-P,IHC-F, WB
https://www.biolegend.com/en-us/products/purified-anti-mouse-cd11c-antibody-1817 Armenian Hamster Mouse IF, Flow Cyt.,IHC,IP,CyTOF
https://www.biolegend.com/en-us/products/purified-anti-mouse-cd4-antibody-484 Rat Mouse IF, Flow Cyt.,IHC, CyTOF
https://www.biolegend.com/en-us/products/purified-anti-mouse-il-17a-antibody-1634 Rat Mouse ELISA,CyTOF
https://www.jacksonimmuno.com/catalog/products/212-035-082, Mouse Rat WB, IHC, ELISA
https://www.jacksonimmuno.com/catalog/products/127-035-160, Goat Hamster WB, IHC, ELISA

In vivo:
https://bioxcell.com/invivomab-anti-mouse-ctla-4-cd152-be0164 Unkown Mouse In vivo, WB
https://bioxcell.com/invivomab-anti-mouse-pd-1-cd279-be0146 Syrian Hamster Mouse In vivo
https://bioxcell.com/invivomab-rat-igg2a-isotype-control-anti-trinitrophenol-be0089 Rat In vivo
https://www.biolegend.com/en-us/products/ultra-leaf-purified-anti-mouse-il-17a-antibody-16817 Rat Mouse In vivo
https://www.leinco.com/p/anti-mouse-ly-6g-purified-functional-grade-gold/ Rat Mouse In vivo, CyTOF,FC,IHC FF,WB
https://www.prospecbio.com/il-17_mouse

Ex vivo:
https://www.biolegend.com/en-us/products/purified-anti-human-il-17a-antibody-4442 Mouse Human ICFC,IF,Flow Cyt.
https://bioxcell.com/invivosim-anti-human-pd-1-nivolumab-biosimilar Human Flow Cyt.,WB,IHC
https://bioxcell.com/invivosim-anti-human-ctla-4-ipilimumab-biosimilar Human Flow Cyt.,WB,ELISA

FACS:
https://www.biolegend.com/en-us/products/percp-cyanine5-5-anti-mouse-cd45-antibody-4264 Rat Mouse Flow Cyt.
https://www.biolegend.com/en-us/products/fitc-anti-mouse-cd3-antibody-45 Rat Mouse Flow Cyt.
https://www.biolegend.com/en-us/products/pacific-blue-anti-mouse-cd4-antibody-2855 Rat Mouse Flow Cyt.
https://www.biolegend.com/en-us/products/brilliant-violet-510-anti-mouse-cd8a-antibody-7992 Rat Mouse Flow Cyt.
https://www.biolegend.com/en-us/products/alexa-fluor-647-anti-human-mouse-granzyme-b-antibody-6067 Mouse Human,Mouse Flow Cyt.

https://www.biolegend.com/en-us/products/percp-cyanine5-5-anti-mouse-cd45-antibody-4264 Rat Mouse Flow Cyt.
https://www.biolegend.com/en-us/products/pacific-blue-anti-mouse-human-cd11b-antibody-3863 Rat Mouse, Human, Cynomolgus, Rhesus Flow Cyt.
https://www.biolegend.com/en-us/products/alexa-fluor-488-anti-mouse-cd11c-antibody-2702 Armenian Hamster Mouse Flow Cyt.
https://www.biolegend.com/en-us/products/alexa-fluor-647-anti-mouse-ly-6c-antibody-4897 Rat Mouse Flow Cyt.
https://www.biolegend.com/en-us/products/pe-anti-mouse-ly-6g-antibody-4777 Rat Mouse Flow Cyt.

Eukaryotic cell lines

Policy information about cell lines and Sex and Gender in Research

Cell line source(s)

The human melanoma cell lines WM983B, 451Lu, and WM9 were obtained from the Wistar Institute.
The mouse melanoma cell lines derived from the MT/ret spontaneous melanoma model (CM and LN, [reference 20]) were obtained from Prof. Iris Helfrich (University Hospital Essen, Germany).
The mouse melanoma cell line YUMM1.7 (CRL-3362) was purchased from ATCC and the YUMMER1.7 (clone D4) cell line was purchased from Merck.

Authentication

Cell line identity was confirmed by PCR-based DNA fingerprinting at the Department of Pathology of the University Hospital Essen.

Mycoplasma contamination

All cell lines were regulalry monitored and tested negative for mycoplasma contamination.

Commonly misidentified lines
(See ICLAC register)

No misidentified cell line was used in this study.

Animals and other research organisms

Policy information about studies involving animals; ARRIVE guidelines recommended for reporting animal research, and Sex and Gender in Research

Laboratory animals

8 weeks old female C57BL/6N or C57BL/6J mice were used for experiments. All mice were housed in rooms maintained at a constant temperature of 22°C and 45-65% humidity with a 12 hour light cycle.
Animals were allowed food and water ad libitum.

Wild animals

The study did not involve wild animals.

Reporting on sex

Female mice were used in this study due to mitigated immune response to ICI therapies in male mice (according to several published reports (references: doi.org/10.3181/00379727-127-32768, doi.org/10.1016/j.bbi.2021.08.225))

Field-collected samples

The study did not involve field-collected samples.

Ethics oversight

All animal experiments were performed in accordance with institutional and national guidelines and regulations. Ethical approval was provided by the local state authority Landesamt für Natur, Umwelt und Verbraucherschutz Nordrhein-Westfalen – LANUV NRW in compliance with the German animal protection law (reference number 81-02.04.2018.A202).

Note that full information on the approval of the study protocol must also be provided in the manuscript.

Flow Cytometry

Plots

Confirm that:

☒ The axis labels state the marker and fluorochrome used (e.g. CD4-FITC).

☒ The axis scales are clearly visible. Include numbers along axes only for bottom left plot of group (a 'group' is an analysis of identical markers).

☒ All plots are contour plots with outliers or pseudocolor plots.

☒ A numerical value for number of cells or percentage (with statistics) is provided.

Methodology

Sample preparation	LegendPlex:<br>Manufarurer's recommended protocol was followed. In brief, plasma samples were incubated with APC beads conjugated with the antibody of interest (bead panels are specified in the Materials and Methods). Afterwards biotinylated detection antibodies (SA-PE) were applied.<br><br>Flow cytometry of murine samples:<br>Tissues were digested using the Mouse Tumor Dissociation Kit (Miltenyi) on the GentleMACS device (Miltenyi) according to the manufacturer’s instruction. Red blood cell lysis buffer (BioLegend) was used to remove red blood cells. After washing with PBS, cells were incubated with TruStain fcX anti mouse CD16/32receptor blocking agent (BioLegend) diluted in Cell Staining Buffer (BioLegend) for 20 min at 4°C. After washing Zombie NIR cell viability dye (1:2000, Biolegend) was added and incubated for 20 min at 4°C. To assess immune cell composition the following antibodies were added for 30 min at 4°C for lymphocytes: anti-CD45 PerCP Cy5.5 (30-F11, 1:100), anti-CD3 FITC (17A2, 1:100), anti-CD4 PB (RM4-5, 1:100) anti-CD8a BV 510 (53-6.7, 1:100), anti-Granzyme B AF 647(GB11, 1:100) ; for macrophages: anti-CD45 PerCP Cy5.5 (30-F11, 1:100), anti-CD11B PB (M1/70, 1:100), anti-CD11C AF 488 (N418, 1:100), anti-Ly6C AF 647 (HK1.4, 1:100), and anti-Ly6G PE (1A8, 1:100), all from Biolegend. Granzyme B was added after surface staining was complete, and after a fixation/permeabilization (Fixation Buffer: BioLegend Cat #420001, 10x Intracellular Staining Perm Wash Buffer, BioLegend Cat #421002).<br>Subsequently, samples were washed twice before data acquisition on BD Aria III flow cytometer. Gating strategy is shown in Extended Figure 3b.
Instrument	FACSAria™ III (BD) machine with nozzle size 70 µm was used.
Software	LegendPlex:<br>Mean fluorescence intensity (MFI) values were recorded from the LEGENDplex™ analysis software (version 2021. 07.01, https://legendplex.qognit.com) and cytokine concentrations (pg/ml) were interpolated from 5-parameter logistic (5PL) non-linear curve model using separate standard curve for each cytokine.<br>Flow cytometry of murine samples:<br>Samples acquisition was done on FACS Diva v.9.0.1. software (BD) and analysis was carried out in FlowJo v. 10.8.1.
Cell population abundance	MACS sorted T cell and neutrophil cellular abundancy was confirmed by flow cytometry and purity above 90% was accepted for downstream applications.
Gating strategy	Cells were gated on forward and side scatter parameters (FSC/SSC), then dead cells (Zombie APC Cy7 positive) were excluded. Immune cells were gated based on CD45 positivity, then lymphocytes were subsequently gated on CD3, then on CD4 or CD8 positive populations. Granzyme B was gated on CD8 positive cells. Neutrophils were gated from the CD45 positive population based on Cd11b/Ly6G double positive cells.

☒ Tick this box to confirm that a figure exemplifying the gating strategy is provided in the Supplementary Information.
