# Supplementary figures and images for: Interleukin 17 signaling supports clinical benefit of dual CTLA-4 and PD-1 checkpoint inhibition in melanoma
Source: Nat Cancer. 2023 Jul 31;4(9):1292–308. doi: 10.1038/s43018-023-00610-2 (PMC10518254; doi:10.1038/s43018-023-00610-2)

Figure 2g

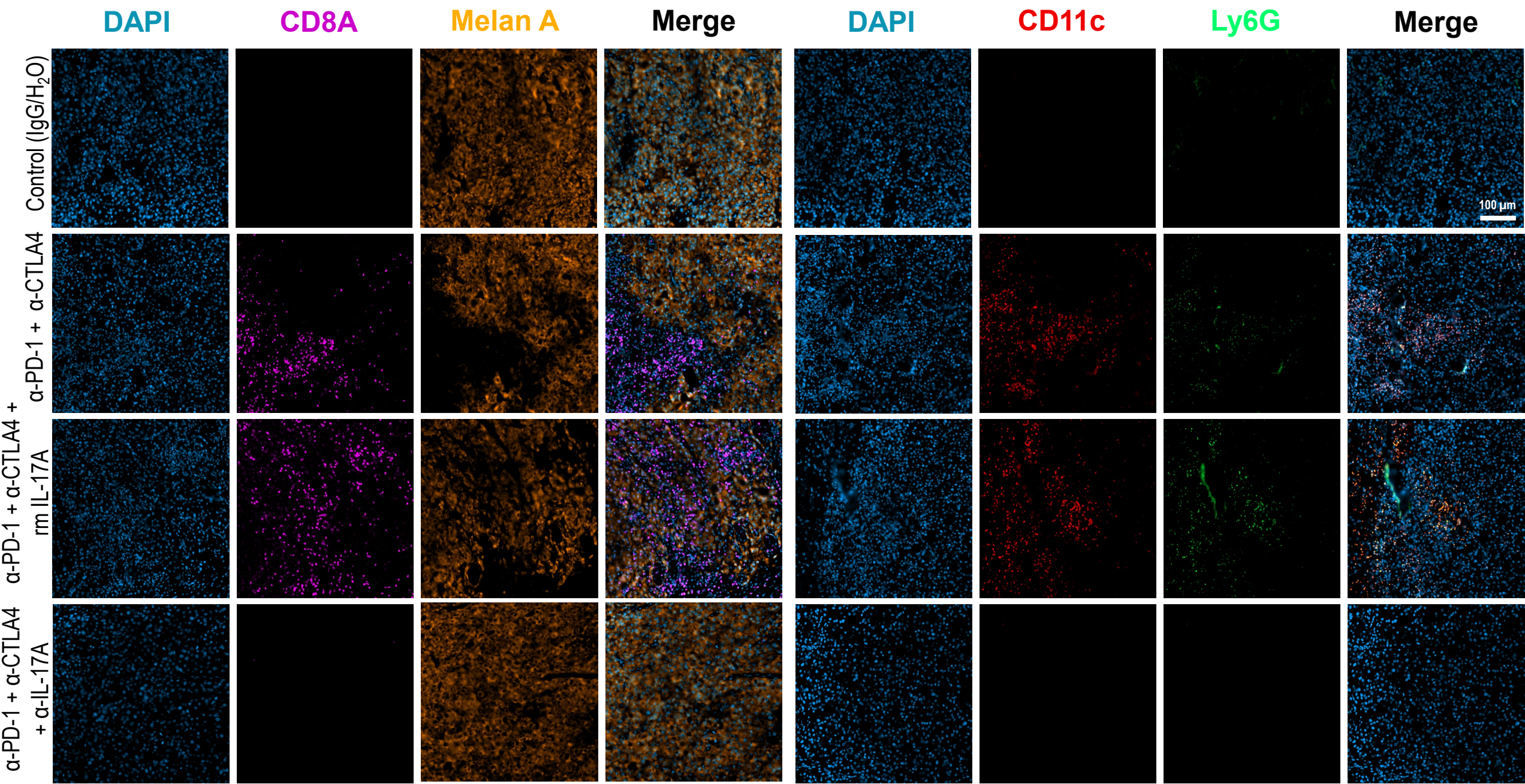

Figure S2b

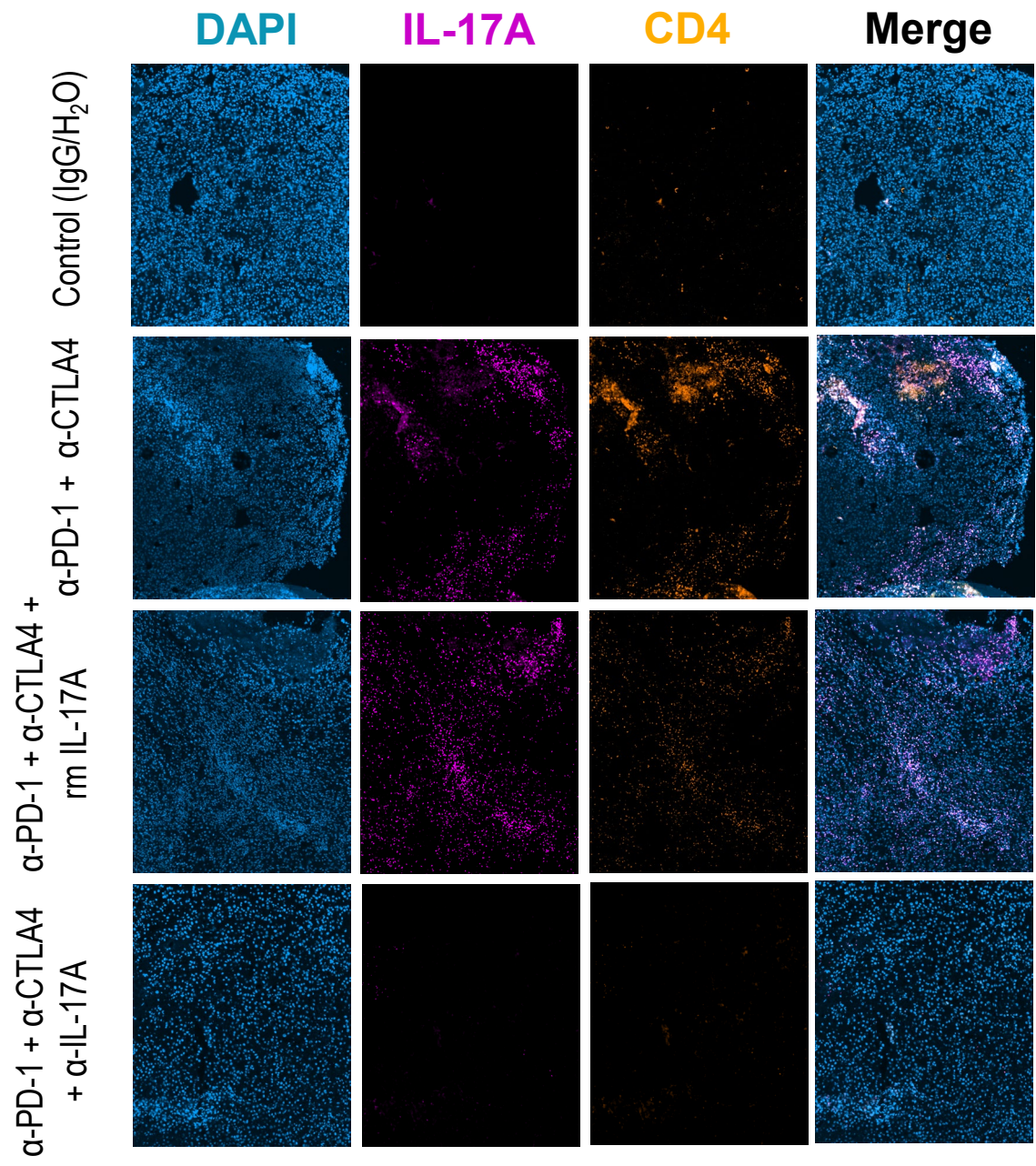

Supplement: Supplementary file 5 — Imaging source data. [file 43018_2023_610_MOESM5_ESM.pdf]
